# Comparative chloroplast genomics of 24 species shed light on the genome evolution and phylogeny of subtribe Coelogyninae (Orchidaceae)

**DOI:** 10.1186/s12870-023-04665-2

**Published:** 2024-01-05

**Authors:** Lin Li, Qiuping Wu, Junwen Zhai, Kunlin Wu, Lin Fang, Mingzhi Li, Songjun Zeng, Shijin Li

**Affiliations:** 1 Key Laboratory of South China Agricultural Plant Molecular Analysis and Genetic Improvement, Guangdong Provincial Key Laboratory of Applied Botany, South China Botanical Garden, Chinese Academy of Sciences, Guangzhou, 510650 China; 2 University of Chinese Academy of Sciences, Beijing, 100049 China; 3 Key Laboratory of National Forestry and Grassland Administration for Orchid Conservation and Utilization at College of Landscape Architecture, Fujian Agriculture, Fujian Agriculture and Forestry University, Fuzhou, 350002 China; 4 Guangzhou Bio & Data Biotechnology Co., Ltd, Guangzhou, 510555 China; 5 Key Laboratory of Plant Resources Conservation and Sustainable Utilization, South China Botanical Garden, Chinese Academy of Sciences, Guangzhou, 510650 China

**Keywords:** Coelogyninae, Chloroplast genome, Comparative analysis, Phylogeny

## Abstract

**Background:**

The orchids of the subtribe Coelogyninae are among the most morphologically diverse and economically important groups within the subfamily Epidendroideae. Previous molecular studies have revealed that Coelogyninae is an unambiguously monophyletic group. However, intergeneric and infrageneric relationships within Coelogyninae are largely unresolved. There has been long controversy over the classification among the genera within the subtribe.

**Results:**

The complete chloroplast (cp.) genomes of 15 species in the subtribe Coelogyninae were newly sequenced and assembled. Together with nine available cp. genomes in GenBank from representative clades of the subtribe, we compared and elucidated the characteristics of 24 Coelogyninae cp. genomes. The results showed that all cp. genomes shared highly conserved structure and contained 135 genes arranged in the same order, including 89 protein-coding genes, 38 tRNAs, and eight rRNAs. Nevertheless, structural variations in relation to particular genes at the IR/SC boundary regions were identified. The diversification pattern of the cp. genomes showed high consistency with the phylogenetic placement of Coelogyninae. The number of different types of SSRs and long repeats exhibited significant differences in the 24 Coelogyninae cp. genomes, wherein mononucleotide repeats (A/T), and palindromic repeats were the most abundant. Four mutation hotspot regions (*ycf1a*, *ndhF-rp132*, *psaC-ndhE*, and *rp132-trnL*) were determined, which could serve as effective molecular markers. Selection pressure analysis revealed that three genes (*ycf1a*, *rpoC2* and *ycf2* genes) might have experienced apparent positive selection during the evolution. Using the alignments of whole cp. genomes and protein-coding sequences, this study presents a well-resolved phylogenetic framework of Coelogyninae.

**Conclusion:**

The inclusion of 55 plastid genome data from a nearly complete generic-level sampling provide a comprehensive view of the phylogenetic relationships among genera and species in subtribe Coelogyninae and illustrate the diverse genetic variation patterns of plastid genomes in this species-rich plant group. The inferred relationships and informally recognized major clades within the subtribe are presented. The genetic markers identified here will facilitate future studies on the genetics and phylogeny of subtribe Coelogyninae.

**Supplementary Information:**

The online version contains supplementary material available at 10.1186/s12870-023-04665-2.

## Introduction

The tribe Arethuseae Lindl. belonging to the subfamily Epidendroideae can be divided into two monophyletic lineages, corresponding to the subtribes Coelogyninae Benth. and Arethusinae Benth. [1–5]. As one of the most diverse and economically important group within the tribe, Coelogyninae in its well-known delimitation comprises 22 genera with approximately 740 species [2, 4, 5] and is widespread in tropical Asia and the Pacific. Many species of Coelogyninae are horticultural plants with great ornamental value, while others have long been used in traditional medicine practices due to their nutritious or medical efficacy [6]. Although it is generally accepted as a monophyletic group, the circumscriptions of some Coelogyninae genera are not well resolved. Previous phylogenetic studies on the subtribe Coelogyninae were based on either traditional morphological characters or only a few loci, which may indicate some limitations. The phylogenetic relationships of the major clades in the Coelogyninae group, particularly those of *Coelogyne* and its related genera remain unclear. Gravendeel et al. [7] delimited *Coelogyne* in the strict sense by including *Neogyna* and *Pholidota* species and removing those species with hairy ovaries and ovate-oblong petals. In contrast, Chase et al. [8] expanded the genus by transferring the former 14 genera into *Coelogyne s.l.*, which comprises about 550 species. It is worth noting that Coelogyninae has been circumscribed to consist of only eight genera after this merging.

Compared with relatively short DNA fragments, the chloroplast (cp.) genomes in vascular plants contain more abundant informative sites. Chloroplast genomes have been increasingly used for phylogenetic reconstruction and genome-wide patterns of nucleotide polymorphism in the family Orchidaceae. Insights gained from whole cp. genome data have greatly improved our understanding of the evolution and diversification of Orchidaceae [9, 10]. Over the past few years, phylogenomic analyses and comparative chloroplast genomics have been conducted in some genera of subtribe Coelogyninae, e.g., *Bletilla*, *Thuniopsis* and *Pholidota* [11–13]. These phylogenomic studies have proven to be effective in improving phylogenetic resolution. For example, Li et al. [13] initially probed the structural patterns of *Pholidota* plastomes and provided new insight into the phylogenetic relationships among *Pholidota* and its related genera using the whole-genome data. So far, however, the deep phylogenetic relationships and genome-wide patterns within the subtribe Coelogyninae have not been thoroughly investigated. The intergeneric and infrageneric classification and relationships within the subtribe remain controversy.

This study represents the most comprehensive taxonomic sampling of Coelogyninae thus far using complete plastomes. We newly sequenced 15 cp. genomes of Coelogyninae species and retrieved nine previously published cp. genomes for comparative and phylogenetic analyses. Here, in order to better show the systematic positions of *Coelogyne* and its related genera, the traditional concept of each genus was adopted.

The major objectives of this study were: (1) to investigate the characteristics and evolutionary patterns of cp. genomes among species in Coelogyninae; (2) to detect variation and mutation hotspots among these cp. genomes; (3) to explore the phylogenetic relationships among the major clades of Coelogyninae.

## Results

### Plastome features of Coelogyninae species

**Fig. 1:**
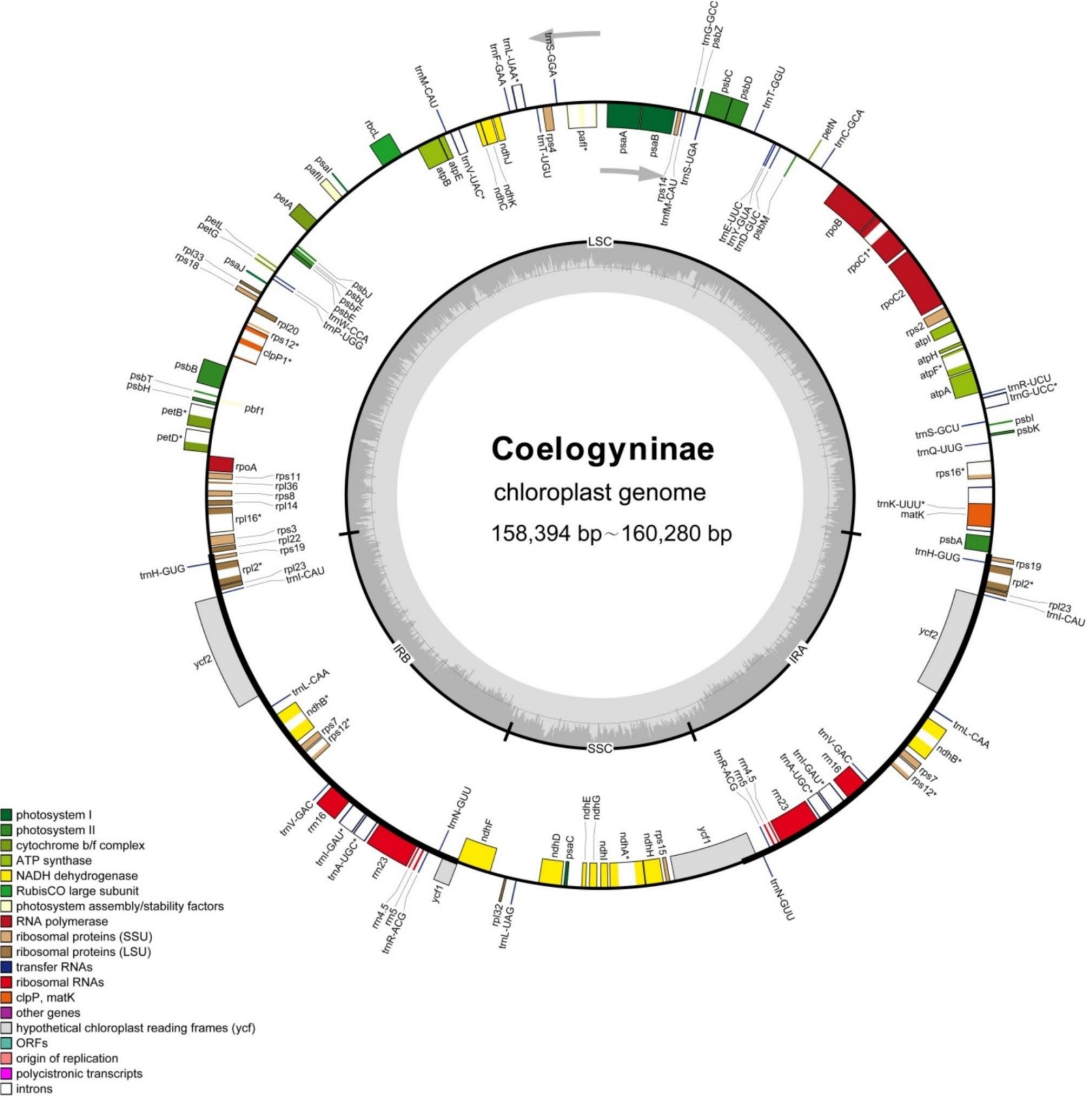
Chloroplast genome organization and characteristics of Coelogyninae. Genes drawn inside and outside of the circle are transcribed in clockwise and counterclockwise directions separately. Darker and lighter gray area in the inner circle corresponds to the GC and AT content, respectively. Genes belonging to different functional groups are color-coded in the outmost circle

**Fig. 2:**
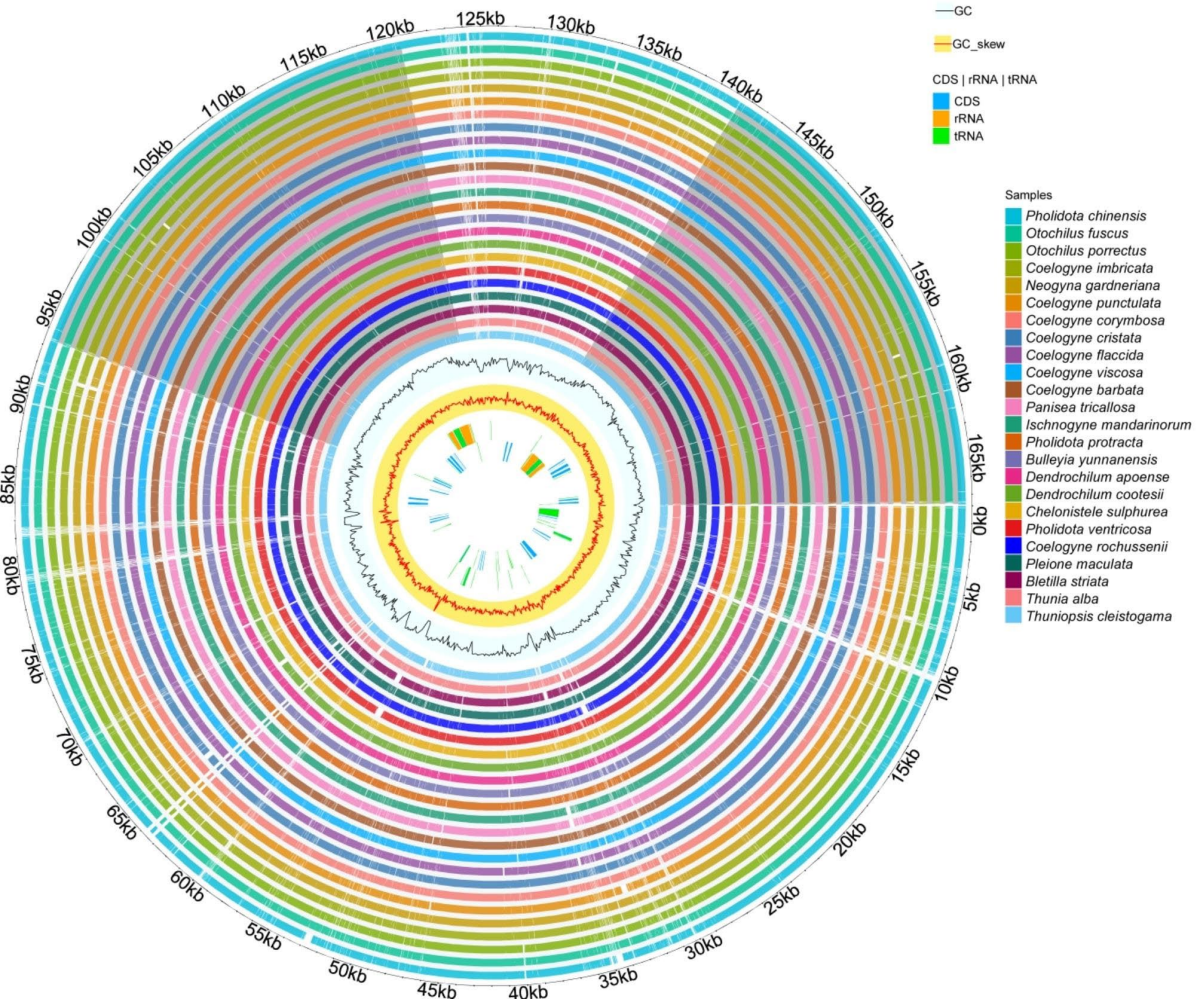
Graphical map of circular genomes retrieved by Gview, providing the overall visualization of the 24 Coelogyninae plastomes. From the inside to the outside, the positions of CDS (blue), rRNA (orange) and tRNA (green) genes on both the forward and reverse strand, followed by GC skew (yellow, red lines), GC content (cerulean, black lines) are shown in sequence. The outer circle indicates the genome size in kbp. The remaining circles display BLAST comparisons of plastome sequences. The innermost circle represents the reference genome of *Thuniopsis cleistogama.* The similar and divergent locations in the plastomes are shown in continuous and interrupted track lines, respectively. The lightly screened area stretching from the inner toward the outer circle indicates different regions with large sequence divergences

The complete chloroplast genomes newly obtained from the 15 Coelogyninae species, plus previously sequenced nine species were very similar in the overall structure, gene content, order and GC content (Figs. 1 and 2; Additional file 1: Fig. S1). The lengths of the 24 Coelogyninae plastomes ranged between 158,394 bp (*Pleione maculata*) and 160,280 bp (*Chelonistele sulphurea*). All of the cp genomes possessed the typical four conjoined structures, including a pair of IRs (26,489–26,796 bp) separated by one LSC (86,603–88,206 bp) and one SSC (18,499–18,885 bp) region. The GC content in the IR regions (43.18–43.34%) was higher than those in the LSC (34.83–35.27%) and SSC (29.9–30.58%) regions (Additional file 2: Table S1). Each of the cp genome comprised 135 genes, including 89 protein-coding genes (PCGs), 38 tRNAs and eight rRNAs. There were 80 genes (59 PCGs and 21 tRNA genes) in the LSC region, 11 genes (10 PCGs and one tRNA gene) in the SSC region, 20 genes included two copies because of their location in the IR regions, including eight protein-coding genes (*rpl2*, *rpl23*, *rps7*, *rps12*, *rps19*, *ycf2*, *ycf15*, and *ndhB*), four rRNA genes (*rrn16*, *rrn23*, *rrn4.5* and *rrn5*), and eight tRNA genes (*trnA*-*UGC*, *trnH*-*GUG*, *trnI*-*CAU*, *trnI*-*GAU*, *trnL*-*CAA*, *trnN*-*GUU*, *trnR*-*ACG*, and *trnV*-*GAC*). Among these genes, 19 genes contained two exons, including 11 coding genes (*atpF*, *ndhA*, two *ndhB*, *petB*, *petD*, *rpl2*, *rpl16*, *rpoC1*, *rps12* and *rps16*) and eight tRNA genes (two *trnA-UGC*, *trnG*-*UCC*, two *trnI*-*GAU*, *trnK*-*UUU*, *trnL*-*UAA* and *trnV*-*UAC*). Four coding genes (two *rps12*, *clpP1* and *paf1*) contained three exons. In all these plastomes, the gene *ycf1* crossed the SSC and IRA junction. The SSC and IRB boundaries were positioned in the genes *ycf1*b and *ndhF*. The *rps12* gene was recognized as a trans-spliced gene, with 5’-end exon located in the LSC region and two 3’-end exons located in IR regions (Fig. 1).

The graphical map of circular genomes was generated by GView to assess sequence variations among the 24 chloroplast genomes in Coelogyninae (Fig. 2). In all the examined plastomes, sequences in the LSC and SSC regions varied greatly across different genera and species. The genome comparison revealed that the sequences in the two IR regions were less divergent than those of the LSC and SSC regions. The intergenic regions exhibited higher divergence than the coding regions.

### Identification of SSRs and long repetitive sequences

**Fig. 3:**
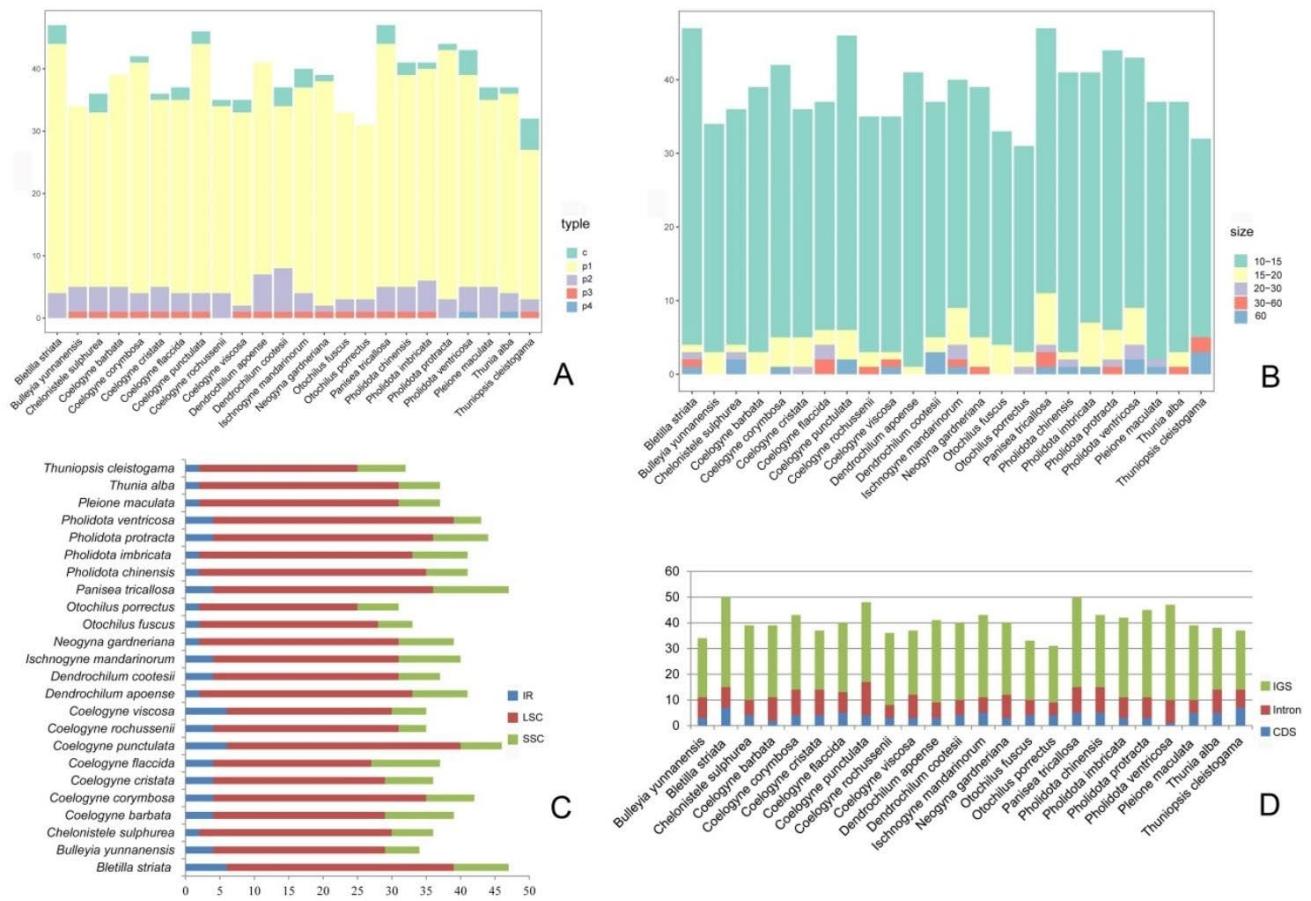
Repeats in the 24 Coelogyninae plastomes. (**A**) Number of different SSRs types (p1–p4 indicate mono-, di-, tri-, and tetranucleotide, respectively; c indicates compound SSRs); (**B**) Number of repeats by length; (**C**) Frequency of SSRs in the LSC, IR, SSC region; (**D**) Frequency of SSRs in the intergenic regions (IGS), protein-coding (CDS) genes and introns

Simple sequence repeats (SSRs) or microsatellites, consisting of short DNA motifs (1–6 bp in length) are specific, highly polymorphic and informative genetic markers and are widely distributed in the genomes. In this study, we examined the SSRs among the 24 Coelogyninae cp. genomes and analyzed their distribution and frequency in different genomic regions. The SSR screening resulted in the identification of 1,014 SSRs, ranged from 31 (*Otochilus porrectus*) to 53 (*Bletilla striata* and *Panisea tricallosa*) (Fig. 3A). The detailed frequency of SSRs with different repeat motif and number is shown in Additional file 3: Table S2. Of the total 1,013 SSRs, 972 were simple repeat motifs (95.95%) and 41 were present in compound formation (4.05%). Among the SSRs, the highest proportion was represented by mononucleotide (p1) repeats, with a proportion of 84.11% and dinucleotide (p2) repeats accounted only for 9.77%. The trinucleotide (p3) and tetranucleotide (p4) repeats were rather rare, accounting for 1.87% and 0.2%, respectively. We also detected the frequency of motif composition in these genomes. The most abundant mononucleotide SSRs were composed primarily of A and T repeat units (96.83%), with only 3.17% composed of C/G. Dinucleotide repeats always consisted of AT and TA (100%), with an obvious A/T bias. For trinucleotide (p3) repeats, TTC repeats were most abundant in many species (89.48%), with ATT repeat motif (5.26%) only detected in *Pholidota imbricata* cp. genome, while TCT repeat motif (5.26%) only occurred in *Chelonistele sulphurea* cp. genome. In addition, tetranucleotide (p4) repeats were not found in most of these genomes, except for one tetranucleotide repeat motif (ATAG) detected in the cp. genome of *Pholidota ventricosa*, and *Thunia alba*, respectively. These results were consistent with previous investigations [12, 13].

The SSRs ranged in size from 10 to 60 bp, with the majority in 10–15 bp length, accounting for 87.71%, followed by 15–20 bp (7.26%), 20–30 bp (1.5%), 30–60 bp (1.39%) and 60 bp (2.14%). The most abundant SSRs in 10–15 bp as well as a wide range of all sizes from 15 to 60 bp were detected in the cp. genomes of *Bl. striata* and *P. tricallosa*, respectively, while in the *T. cleistogama* cp. genome where SSRs preferred lengths were generally longer (30 to 60 bp) in comparison with SSRs in most other cp. genomes (Fig. 3B; Additional file 4: Table S3).

Furthermore, the SSRs in the 24 Coelogyninae cp. genomes were more frequently located in the LSC region (73.54%) than in the SSC region (18.36%), and only a minority (8.1%) was dispersed within the IR regions (Fig. 3C; Additional file 5: Table S4). Likewise, SSRs (70.48%) in these cp. genomes primarily occurred in the intergenic spacer (IGS) regions, with a small portion (19.55%) distributed in introns, while only a few (9.97%) of SSRs was found in CDS regions (Fig. 3D; Table S4).

**Fig. 4:**
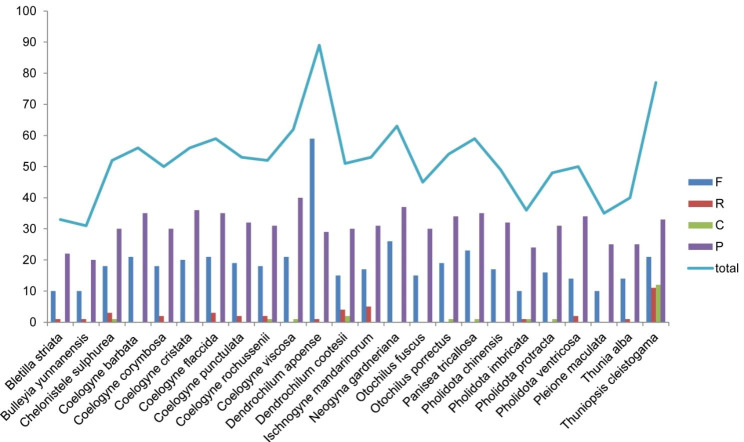
Frequency of four long repeat types: Forward (F), Reverse (R), Complement (C), Palindromic (P) in the 24 Coelogyninae plastomes

Long repetitive sequences were detected with a length ≥ 30 bp and similarity > 90% between two copies. In total, 1,253 long repetitive sequences were detected in the 24 Coelogyninae cp. genomes, including 10–59 forward (F) repeats, 0–11 reverse (R) repeats, 0–12 complementary (C) repeats, and 20–40 palindromic (P) repeats (Fig. 4, Additional file 6: Table S5). Of the four types of long repeats, most of those were palindromic (P) and forward (F) repeats, with percentages of 59.14% and 36.07%, respectively, with complementary (C) and reverse (R) repeats accounted for only 1.68% and 3.11%, respectively.

Among the 24 Coelogyninae cp. genomes, *Dendrochilum apoense* contained the highest number (89) of long repeats, while *Bulleyia yunnanensis* contained the lowest (31). Only five species (*Ch. sulphurea*, *C. rochussenii*, *D. cootesii*, *Ph. imbricata* and *T. cleistogama*) had all four types of repeats (Fig. 4, Additional file 6: Table S5). Of these species, *T. cleistogama* had 11 reverse (R) repeats and 12 complementary (C) repeats, while most other species had only 1–4 reverse repeats and 1–2 complementary repeats. For three types of long repeats detected in 13 cp. genomes, most of which had 1–5 reverse (R) repeats and lacked complementary (C) repeats. Interesting, there was no reverse (R) repeat in the plastomes of four species (*C. viscosa*, *O. porrectus*, *P. tricallosa* and *Ph. protracta*), instead, there was only one complementary (C) repeat detected. The result suggests that long repeats are not only typical and but also representative in these species.

### Comparison of IR/SC boundaries

**Fig. 5:**
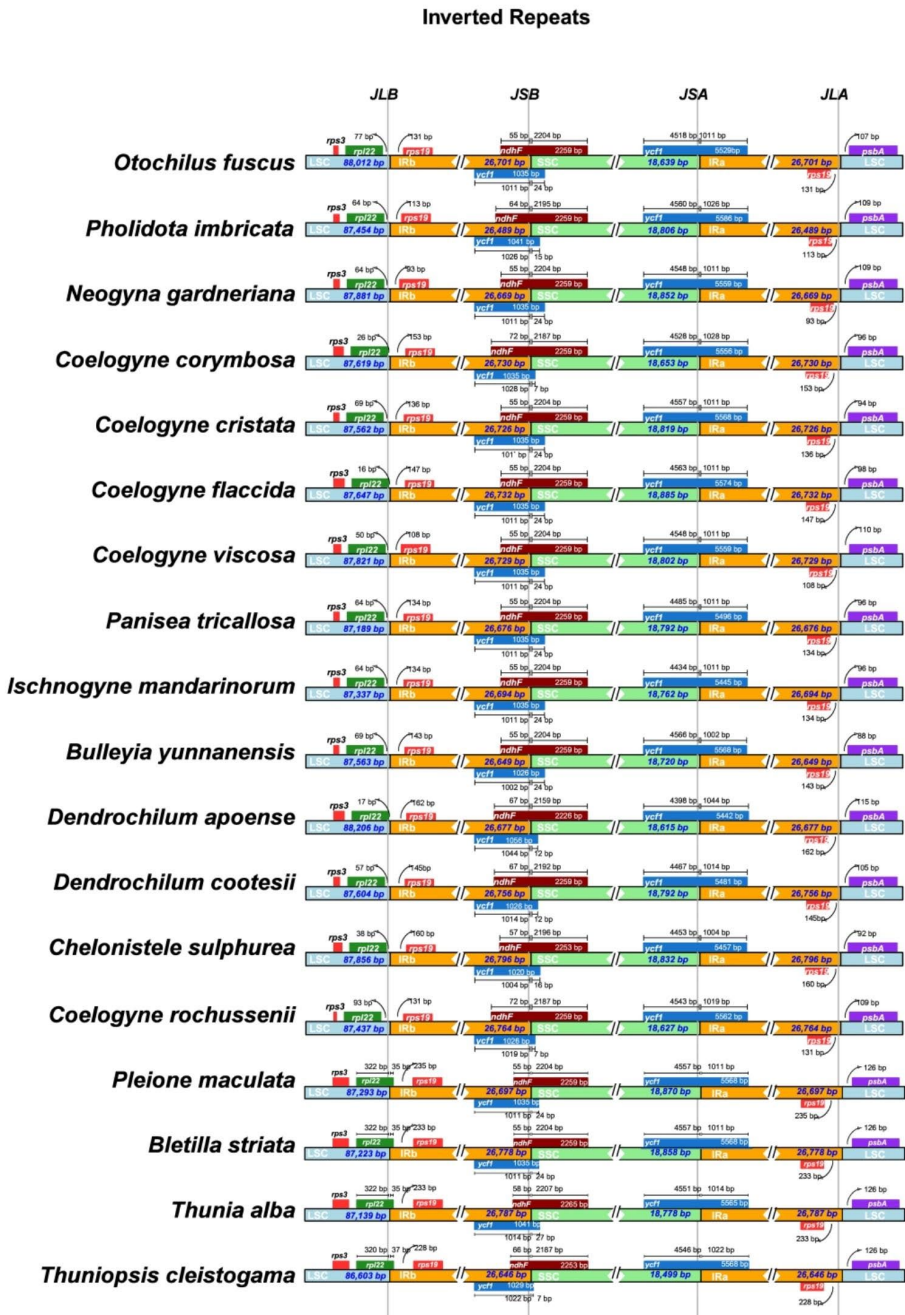
Comparison of the junctions between the LSC/SSC and IR regions among the 18 Coelogyninae chloroplast genomes

The IR/LSC and IR/SSC boundary positions were visualized among the 18 plastomes from the representatives belonging to 13 genera of Coelogyninae. In all these plastomes, gene *rps19* was located near the LSC/IR border, while both genes *ycf1* and *ndhF* were located at the IR/SSC junctions. In addition, overlaps between the *ycf1* and *ndhF* were detected at the IRb/SSC boundaries. Although these plastomes were highly similar regarding their gene content and order, the IR/SC junctions showed substantial differences among the genera and species within the subtribe (Fig. 5).

The SSC/IRa junctions were embedded in the *ycf1* gene, resulting in an incomplete duplication in the IRb region, with the portion located in IRb region varied from 1,002 bp (*Bulleyia yunnanensis*) to 1,044 bp (*Dendrochilum apoense*). The *ndhF* gene spanned over the IRb/SSC junctions, partially overlapping with the duplicated *ycf1*, with the overlapping lengths at the IRB/SSC border varied from 55 to 72 bp. The length of *ndhF* exhibited a high degree of uniformity. The majority of these plastomes had the same length of 2,259 bp, with three exceptions (2,226 bp in *D. apoense*, 2,253 bp in *Chelonistele sulphurea*, and 2,265 bp in *Thunia alba*).

For the 14 Coelogyninae species representing *Coelogyne* and closely related genera, the *rpl22* gene was completely positioned in the LSC region, with the distances from the LSC/IRb border varying from 16 bp (*Coelogyne flaccida*) to 93 bp (*C. rochussenii*). Interestingly, for the rest four Coelogyninae species, *Bletilla striata*, *Pleione maculata*, *Thunia alba*, and *Thuniopsis cleistogama*, the *rpl22* gene spanned the LSC/IRb junction, with the portion located in the IRb region ranged only from 35 to 37 bp.

Furthermore, the IRa/LSC region was located in the intergenic regions of two genes, *rps19* and *psbA*. The gene *rps19* adjacent to the IR/LSC junctions was duplicated in the IR regions, with the distances apart from the LSC/IRb border varying from 93 bp (*Neogyna gardneriana*) to 162 bp (*D. apoense*) for the 14 Coelogyninae species. However, in the four species (*Bl. striata*, *Pl. maculata*, *T. alba*, and *T. cleistogama*), the distances from the border varied little, only from 228 bp (*T. cleistogama*) to 235 bp (*Pl. maculata*). Similarly, the distance from the gene *psbA* to the IRa /LSC boundary ranged from 88 bp (*B. yunnanensis*) to 115 bp (*D. apoense*) for the 14 species, whereas, the distance was the same 126 bp in the four other species.

### Genome comparison and sequence divergence analyses

**Fig. 6:**
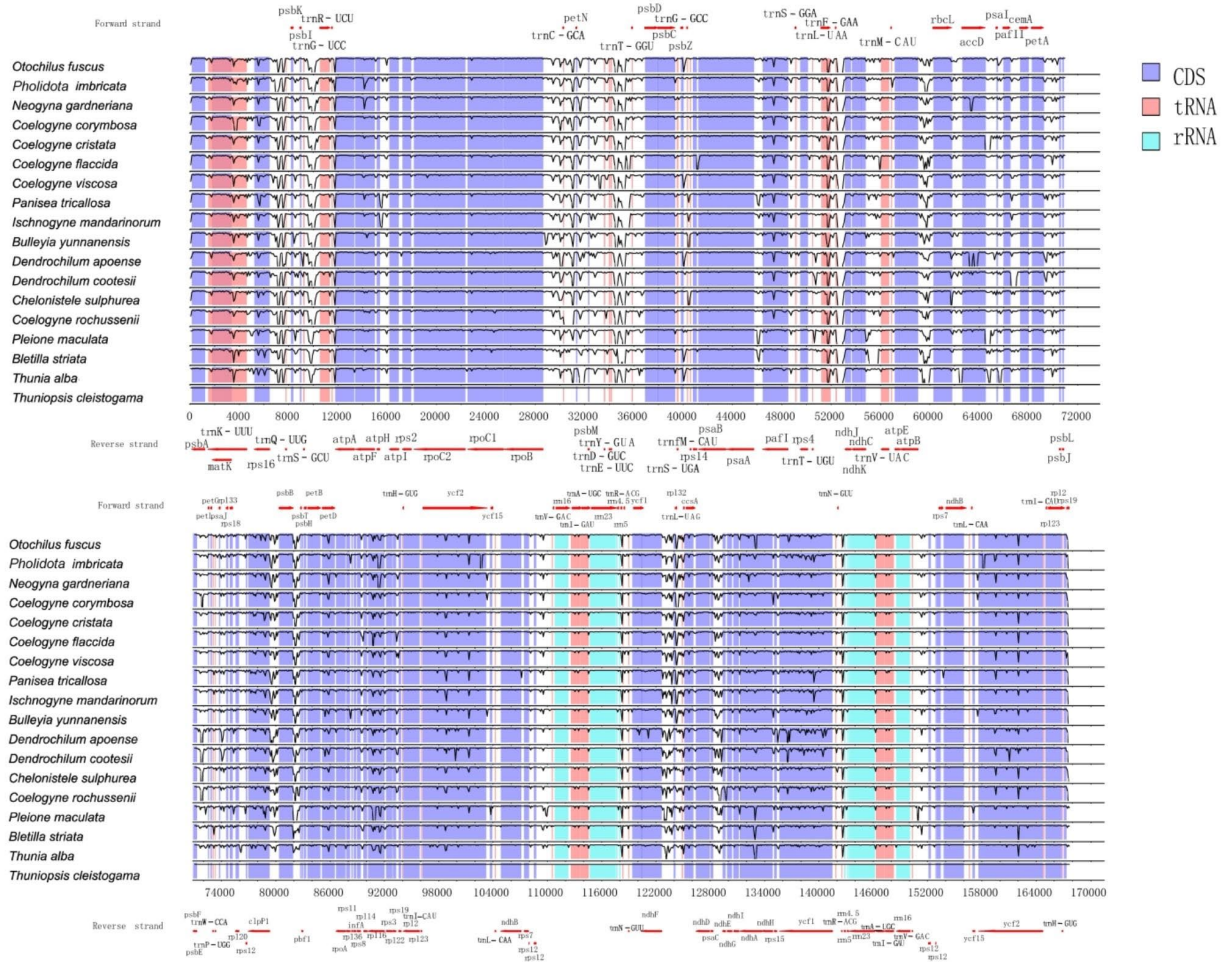
Sequence alignment of the complete plastome sequences of 18 Coelogyninae species with *Thuniopsis cleistogama* cp. genome as a reference. Red arrows up the alignments indicate the direction of the gene. Blue and white correspond to coding regions and non-coding regions, respectively

Taking the annotated *Thuniopsis cleistogama* (OL809660) genome sequence as a reference, mVISTA was carried out to ascertain the divergent regions in the multiple alignments of 18 Coelogyninae cp. genomes representing 13 genera (Fig. 6). Higher degree of variations primarily occurred in the IGS regions, such as *rps16-trnG-UCC*, *rpoB-psbD*, *psbK-psbI*, *rps12*-*trnV-GAC*, *ndhF*-*rp132*, *rp132*-*trnL*-*UAG*, *atpB*-*rbcL*, *atpF*-*atpH*, *atpH*-*atpI*, *trnE*-*UUC*-*trnT-GGU*, *psaA*-*pafI* and *psaC*-*ndhE*. More variations were also identified in the intron-containing genes, such as *rpoC1*, *ycf2*, *ccsA*, *ndhF* and *ycf1*. Most of the genes in CDS region were relatively well conserved, except for the sequence variations in some genes, e.g., *rps16*, *atpF*, *pafI*, *accD*, *clpP1*, *petD*, *rpl16* and *ndhA*. In contrast, the rRNA genes of these species were highly conserved.

Additionally, the nucleotide variability (Pi) values in the LSC, SSC, and IR regions were calculated separately among the 24 Coelogyninae chloroplast genomes. The SSC regions showed the highest nucleotide diversity (Pi = 0.01747), followed by the LSC region (Pi = 0.01055), while IR regions showed the lowest nucleotide diversity (Pi = 0.00256). The results showed that the LSC and SSC regions were more divergent than the IR regions. As expected, the average Pi value in the non-coding regions (0.01113) exhibited comparably higher divergence levels when compared to that in the coding regions (0.00641).

**Fig. 7:**
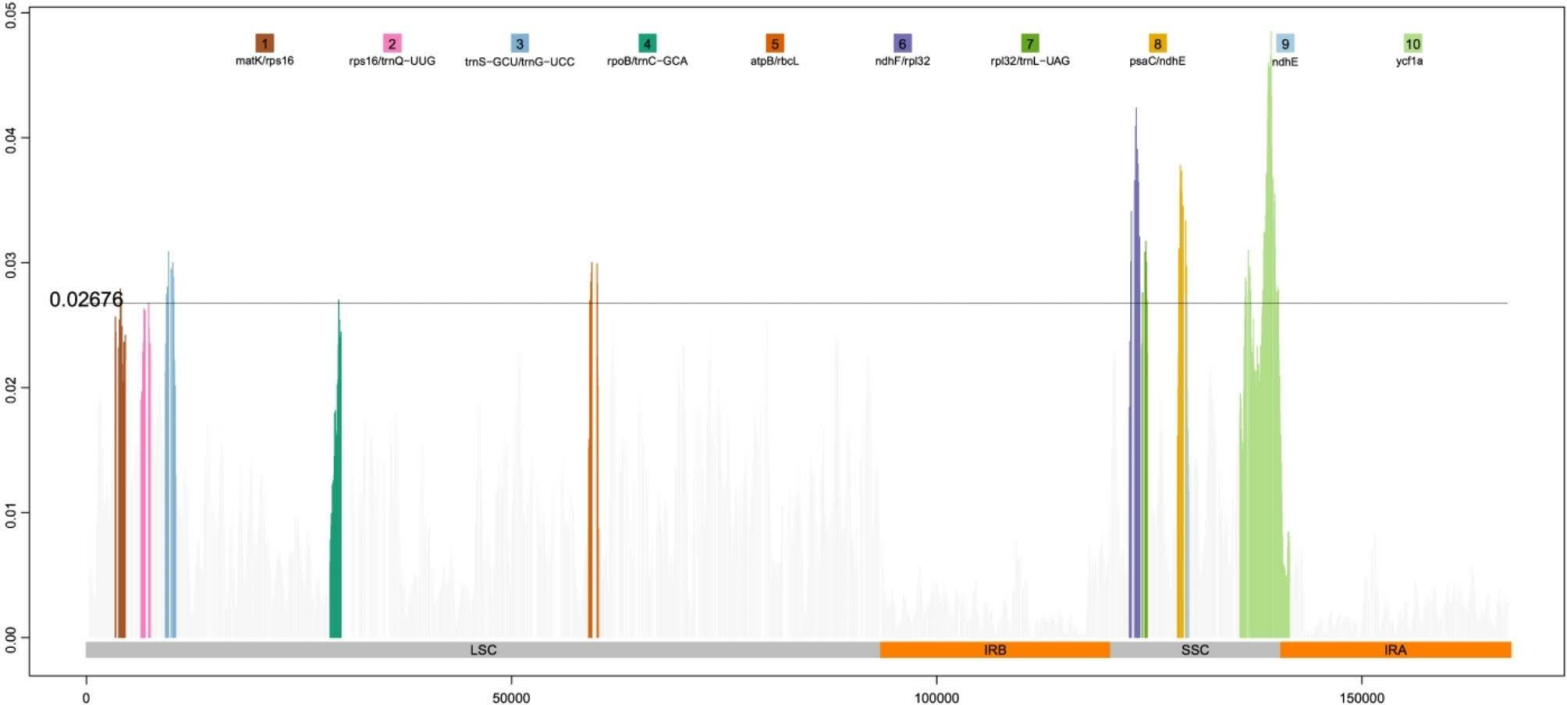
Sliding-window analysis based on the whole cp. genomes of 24 Coelogyninae species. X-axis shows the genomic regions; Y-axis denotes the Pi values

The sliding window analysis revealed ten highly divergent hotspots among the cp. genomes with Pi values ranging from 0.02676 to 0.04849 (Fig. 7). Among the ten regions, five regions: *trnS*-*trnG* (0.03086), *atpB*-*rbcL* (0.02999), *matK*-*rps16* (0.02788), *rps16*-*trnQ* (0.02676) and *rpoB*-*trnC* (0.02701) were located in the LSC region, and four regions: *ndhF*-*rp132* (0.04239), *psaC*-*ndhE* (0.03781), *rp132*-*trnL* (0.03169) and *ndhE* (0.02739) were located in the SSC region, and only *ycf1a* (0.04849), crossed the SSC-IRa boundary in the coding region.

The region of *ycf1a* showed the highest nucleotide variability (0.04849). Three of the variable intergenic spacer (IGS): *ndhF*-*rp132* (0.04239), *psaC*-*ndhE* (0.03781) and *rp132*-*trnL* (0.03169) had markedly higher divergence values (> 0.031). These hypervariable regions or genes were identified as potential molecular markers for further development such as DNA barcoding, molecular phylogenetics and breeding.

### Positive selection sites

We investigated the ratio of non-synonymous to synonymous substitutions (dN/dS) to estimate the selective pressure on 81 common non-redundant protein-coding genes among 20 Coelogyninae species. Positively selected sites under positive selection were detected using codon substitution models. A total of 19 genes with positive selection sites were identified (Additional file 7: Table S6), which were listed as follows: one subunit of the Acetyl-Co A-carboxylase gene (*accD*), one subunit of the ATP-dependent CLP protease gene (*clpP1*), one gene encoding the maturase K (*matK*), one subunit of the rubisco gene (*rbcL*), four genes for a component of the trans locus of an envelope protein (*ycf1a*, *ycf2*, *ycfb* and *ycf15*), one gene for photosystem I subunit (*psaB*), one subunit of ATP synthase gene (*atpB*), six genes for subunits of NADH-dehydrogenase (*ndhA*, *ndhD*, *ndhE*, *ndhF*, *ndhG* and *ndhK*), and three DNA-dependent RNA polymerase genes (*rpoA*, *rpoB* and *rpoC2*).

Among the 19 protein coding genes containing amino acid positive sites, it was found that the *ycf1a* gene located in the IR region harbored the highest number of positive selection sites (113), including 34 significant positive selection sites and 79 extremely significant positive selection sites, followed by *rpoC2* (100) and *ycf2* (96).

### Phylogenetic relationships

**Fig. 8:**
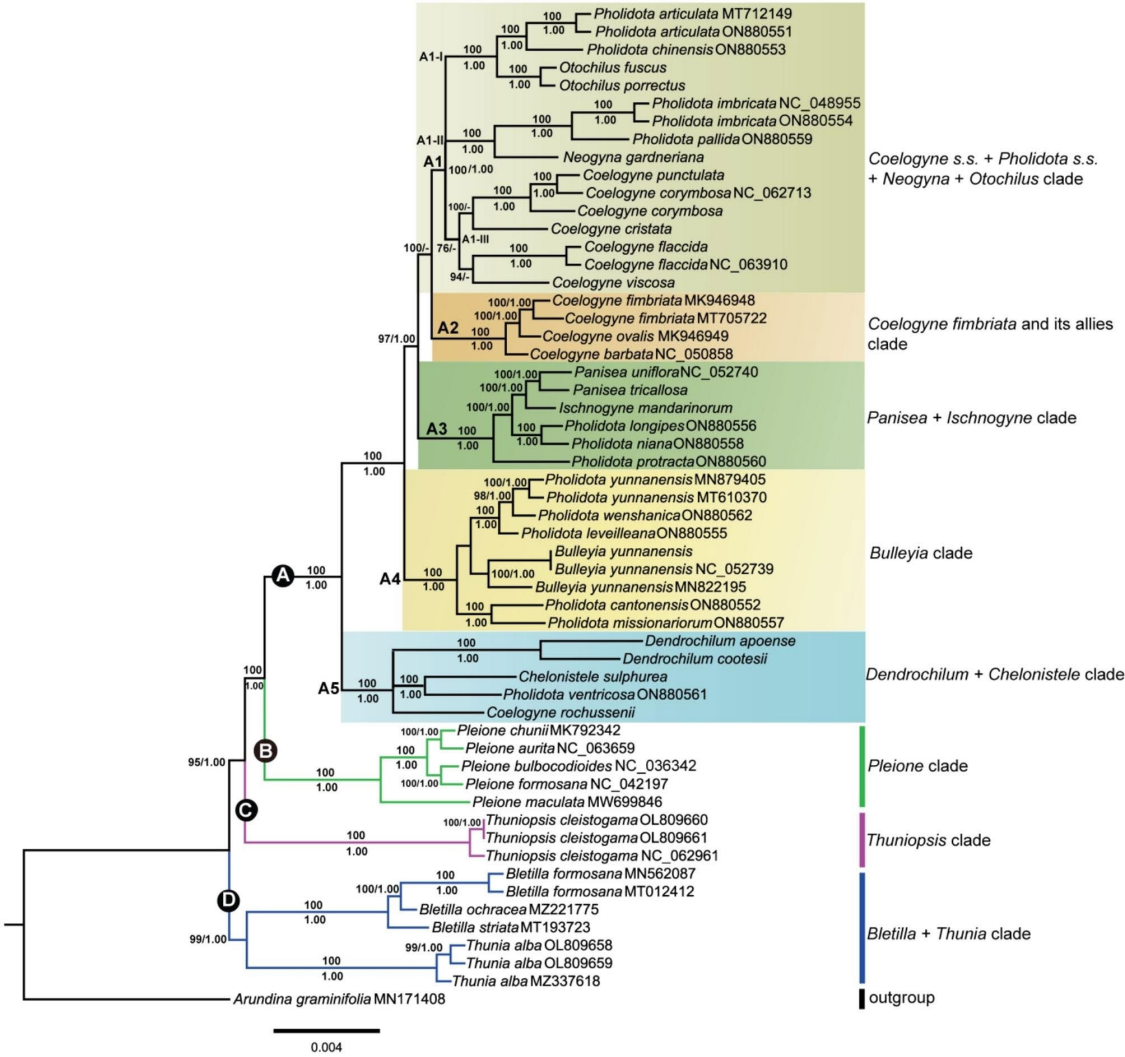
Phylogenetic tree of 42 Coelogyninae species using the maximum likelihood (ML) analysis based on whole chloroplast genomes with *Arundina graminifolia* as outgroup. Clades discussed in the text are signified by Roman numerals. The phylogenetic positions of different clades are highlighted in different colors. Numbers at nodes indicate ML bootstrap values (BS; before the slash) and Bayesian posterior probabilities (PP; after the slash). Dash (–) indicates nodes with PP < 0.5 which is incongruent with ML analysis

Phylogenetic analyses were conducted with the ingroups using a total of 55 accessions, representing 42 species of 13 genera in Coelogyninae. Generally, the topologies of the ML and BI trees constructed with complete cp. genomes (Fig. 8, Additional file 8: Fig. S2) and CDSs (Additional file 9: Fig. S3 and Additional file 10: Fig. S4) were almost identical with only a few exceptions at the shallow nodes. Slightly different tree topologies may be mainly due to the discrepancy in the number of variable sites. Most nodes were maximally supported in the ML tree of Coelogyninae inferred from full plastome sequences. On consideration, we present here the topology resulting from plastome-based ML analysis, with posterior probability (PP) and maximum likelihood bootstrap values (BS) labeled on the tree branches. The positions of representative genera and species in each clade were labelled (Fig. 8).

As seen in Fig. 8, Both ML and Bayesian analyses of the whole plastomes recovered four major monophyletic lineages of Coelogyninae species: clade A consisting species of *Coelogyne* and related genera was strongly supported (BS: 100, PP: 1.0) and further split into several subclades; clade B, *Pleione* clade (BS: 100, PP: 1.0); clade C, *Thuniopsis* clade (BS: 100, PP: 1.0); and clade D, *Bletilla* + *Thunia* (BS: 99, PP: 1.0). *Arundina graminifolia* occurred in a basal position, as an unsupported sister to Coelogyninae.

Clade A was further divided into five highly-supported subclades (A1–A5): A1, composed of a mixed subclade (*Coelogyne s.s.* + *Pholidota s.s.* + *Neogyna* and *Otochilus s.s.*); A2, corresponding to the *C. fimbriata* and its allies clade; A3, *Panisea* + *Ischnogyne* clade; A4, *Bulleyia* clade, and A5, the *Dendrochilum* + *Chelonistele* clade always appeared among the early branching clade.

The monophyletic clade A1 (BS: 100, PP: 1.0), representing the core members of *Coelogyne*, *Neogyna*, *Otochilus* and *Pholidota* respectively, was further resolved to three subclades and each received high supports. The small clade A1-I, including the generic type of *Otochilus*: *O*. *porrectus*, and several sampled species of *Pholidota* (*P. articulata* and *P*. *chinensis*) was recovered as a monophyletic lineage (BS: 100, PP: 1.0); the small clade A1-II comprising the generic type of *Pholidota*: *P*. *imbricata*, its sister *P*. *pallida* and monotypic *Neogyna* was retrieved as monophyletic (BS: 100, PP: 1.0). The type species of *Coelogyne*, *C*. *cristata* was well supported (BS: 100) as a sister to three samples for sect. *Ocellatae* (*C*. *corymbosa* and *C*. *punctulata*), and together formed a moderately supported sister clade A1-III (BS: 76) to three samples for sect. *Flaccidae* (*C*. *flaccida* and *C*. *viscosa*). However, in the BI tree (Additional file 8: Fig. S2), *C*. *cristata* was a close sister (PP: 1.0) to the clade consisting of *Pholidota s.s.* and *Neogyna*. The second clade A2 (BS: 100, PP: 1.0) including four samples for *Coelogyne* sect. *Fuliginosae* (*C*. *fimbriata* and *C*. *ovalis*) and sect. *Elatae* (*C*. *barbata*) formed a well-supported monophyletic lineage. Clade A3 containing two species of *Panisea*, monotypic *Ischnogyne* and three species of *Pholidota* (*P. longipes*, *P. niana* and *P*. *protractas*) was strongly recovered as monophyletic (BS: 100, PP: 1.0) and together referred to as the *Panisea* clade. Clade A4 including monotypic *Bulleyia* and six accessions of five *Pholidota* species (*P*. *cantonensis*, *P*. *leveilleana*, *P*. *missionariuorum*, *P*. *wenshanica* and *P*. *yunnanensis*) were recovered with full support (BS: 100, PP: 1.0), referred to here as the *Bulleyia* clade. Two sampled species of *Dendrochilum*, type species of *Chelonistele*: *Ch. sulphurea* and its sister: *Pholidota ventricosa*, together with *Coelogyne rochussenii*, clustered into the monophyletic clade A5 (BS: 100, PP: 1.0) and referred to as the *Dendrochilum* + *Chelonistele* clade. The five clades A1–A5 were well supported as successive sister groups to each other and relationships among major lineages have received consistent support.

## Discussion

As displayed in Figs. 1 and 2, the 24 Coelogyninae cp. genomes are extremely well conserved with regard to structure, gene composition and order, similar to those previously reported from other species in the subtribe Coelogyninae [11–13]. General characteristics of all 24 Coelogyninae plastomes are shown in Additional file 2: Table S1. Among these cp. genomes, the cp. genome of *Pleione maculata* showed the smallest size (158,394 bp) compared with those of the other Coelogyninae species. Similarly, it also had the smallest LSC and SSC regions (86,603 and 18,499 bp, respectively). The overall GC content detected in these plastomes had less fluctuation, ranging from 37.05 to 37.41%, but varied greatly among different genomes and different genomic regions within a genome. Generally, the IR regions comprised the highest GC content (43.18–43.34%), whereas the SSC region contained the lowest GC content (29.9–30.58%). The GC content values were regular as expected based on AT-rich plastome organization, which were similar to those of most other sequenced angiosperm genomes so far. The IR regions contained consistently higher GC content than that of the SC regions, probably because of four copies of GC-rich rRNA genes (*rrn4.5*, *rrn5*, *rrn16* and *rrn23*) located in the IR regions [14].

The expansion and contraction of IR/SC boundary regions are considered to be common evolutionary events and may cause size variations [15–18]. As seen in Fig. 5, comparison of the IR/SC junction positons among the Coelogyninae plastomes revealed obvious variations. The *rpl22* gene was consistently situated in the LSC region and separated from the LSC/IRb border across the 14 Coelogyninae cp. genomes. However, this gene crossed the LSC/IRb junctions for the cp. genomes of four species, *Bl. striata*, *Pl. maculata*, *T. alba* and *T. cleistogama*. Overall, the IR/SC boundary positions of these four species were more similar than those of the other 14 Coelogyninae species. In most cases, more closely related species are often assumed to have similar responses to environmental changes, so these IR boundary shifts tend to be relatively minor, involving only a small number of genes [19, 20]. The patterns of variation observed in these cp. genomes were generally consistent with the main clades (nodes A, B, C and D, Fig. 8) of Coelogyninae species recovered in the phylogenetic analyses.

Repetitive sequence analysis indicated uneven distribution of polymorphic SSRs across the 24 Coelogyninae plastid genomes with variations in number, size and type of SSR motifs (Fig. 3; Additional file 3–5: Tables S2–S4). Likewise, long repetitive sequences in these genomes also displayed distinct proportion of repeat types (Fig. 4, Additional file 6: Table S5). The accumulation of specific motifs for different SSR types might be the result of selective constraints [21]. Carmona et al. [22] suggested that changes in the quantities and distribution of repetitive DNA sequences are major driving forces of genome evolution and speciation.

To evaluate the sequence divergence level across the 24 Coelogyninae species, we compared the nucleotide diversity in the LSC, SSC and IR regions of the cp. genomes (Fig. 7). The IR regions have lower nucleotide diversity than that of the LSC and SSC regions, possibly due to copy correction between IR sequences caused by gene conversion [23, 24]. As expected, the greatest sequence divergence among the genomes was located in the intergenic areas, which is a common phenomenon in cp. genomes [25]. The dN/dS values varied among groups of different functional genes and provide measures of adaptation or functional constraint in protein-coding genes [26]. Although the majority of protein-coding genes were found to have been subjected to purifying selection, we identified 19 genes that evolved under positive selection (Additional file 7: Table S6). The genes *accD*, *rpoC2*, *ycf1* and *ycf2* have been reported under positive selection in orchid species [9]. Moreover, we found that genes *ycf1a*, *rpoC2* and *ycf2* possessed higher number of positive amino acid sites (113, 100, 96, respectively) within Coelogyninae species. The gene *rpoC2* encodes the subunit of DNA-dependent RNA polymerase gene, and *ycf1* and *ycf2* genes encode unknown function proteins. These genes may play an important role in the adaptation to diverse environments for Coelogyninae species.

Overall, all phylogenetic analyses resulted in largely identical tree topologies of Coelogyninae with the incongruence mainly occurred in the interspecific relationships within clades (Fig. 8; also see Additional file 8: Fig. S2, Additional file 9: Fig. S3, and Additional file 10: Fig. S4). For the whole plastome data, Coelogyninae species fell into four major clades (A, B, C, and D), with clade A consisting of four to five subclades. The monophyly of the four major clades of Coelogyninae represented in this study was well-resolved, with each clade recovered with strong to full support, except for minor interspecific differences. The early diverging Coelogyninae species composed of *Bletillia* + *Thunia* and *Thuniopsis* were successive sister lineages to the clade containing species of *Pleione*, *Coelogyne* and related genera. This relationship was compatible with our former phylogenetic study [5] inferred from the nuclear ITS and two plastid regions.

Presence of storage organs such as rhizomes, succulent stems, tubers, corms or pseudobulbs is important for understanding the adaptations of orchids to survive and thrive in harsh environments. Morphologically, species of *Bletilla*, *Thunia* and *Thuniopsis* all lack heteroblastic pseudobulbs [8, 27]. *Bletilla* species are characterized by having short stems with two to four leaves and irregular subterranean tuberous rhizomes. However, the latter two possess elongated stems with many distichous leaves [4]. For *Thunia* species, they have no subterranean corms or tubers but possess biennial, thick, bamboo-like stems that develop from the previous season’s growth, wither and die during the second season, while *Thuniopsis* has prominent subterranean corms which normally become dormant under stressful conditions [4, 5, 8]. The material of *Aglossorrhyncha*, *Dilochia* and *Glomera* was not available. In our former study, *Dilochia* was moderately supported as a close sister to *Thunia* [5]. Despite its superficial resemblance to *Thunia*, *Dilochia* species can be distinguished by having long-lived, stout but not fleshy stems. Instead, *Aglossorrhyncha* and *Glomera* species can usually be distinguished by more or less rhizomatous, often branching and basally rooting stems [4]. These two genera may also form separate lineages inferred from morphological comparisons and previous molecular data [5, 28, 29]. In contrast to all these species, *Coelogyne* and *Pleione* species have heteroblastic pseudobulbs with one or two apical leaves [4, 8]. Unlike the former, *Pleione* species have only short-lived deciduous pseudobulbs and leaves, and annually renew their pseudobulbs [4], which seem to be an intermediate state of true pseudobulbs. In our phylogenetic analysis (Fig. 8), the sampled species of *Pleione* formed a monophyletic clade B, which was resolved as sister to the clade A containing *Coelogyne* and related species.

The phylogenetic relationships among the major clades inferred by ML and BI analyses were almost congruent. Two sampled species of *Coelogyne* and *Pholidota* clustered into the *Chelonistele* + *Dendrochilum* clade, which was resolved as a distinct and divergent lineage, well separated from the *Coelogyne s.s.* and *Pholidota s.s.* clade. The result was consistent with previous studies using nuclear DNA and partial plastid sequences [5, 7, 30]. It was also clear that three sampled species of *Pholidota* and a well-supported small clade composed of monotypic *Ischnogyne* and *Panisea* species formed a monophyletic clade with maximum support, which we referred to as the *Panisea* + *Ischnogyne* clade. Similarly, monotypic *Bulleyia* and five sampled *Pholidota* species were consistently placed as sister group that together formed the *Bulleyia* clade with full support. The close affinity between *Bulleyia*, *Panisea* and these species of *Pholidota* was also indicated in our former results [13].

Despite these consistencies, topological ambiguities occur in the placement of a few *Coelogyne* species. The plastome-based ML analysis clearly placed *C. cristata* (type species of *Coelogyne*) as a sister to species sampled for section *Ocellatae* (*C*. *corymbosa* and *C*. *punctulata*) with maximal statistical support (100%), which was also highly supported (0.94) by BI analysis using the CDS sequences (Additional file 9: Fig. S3). These species clustered together as a moderately supported sister (76%) to *C*. *flaccida* and *C*. *viscosa* (Fig. 8). In plastome-based BI tree, however, *C*. *cristata* was inferred as sister taxon to a clade comprising *P. imbricata* (type species of *Pholidota*), *P*. *pallida* and *Neogyna gardneriana* (Additional file 8: Fig. S2). In all the analyses, species sampled for sect. *Ocellatae* (*C*. *corymbosa* and *C*. *punctulata*) were always resolved as a monophyletic clade with a high-resolution value. The plastome-based ML analysis showed that this clade was sister to *C. cristata*, whereas, it was clustered within the clade composed of the sampled species for *Coelogyne* sect. *Fuliginosae* (*C*. *fimbriata* and *C*. *ovalis*) and sect. *Elatae* (*C*. *barbata*) in plastome-based BI tree. Furthermore, the phylogenetic positions of *C*. *flaccida* and *C*. *viscosa*, were not consistent across the different datasets.

Based on the available molecular data, the placement of some *Coelogyne* species endemic to southwest China and the Sino-Himalayan Mountains has been unstable, probably because the limited sampling might have influenced the analyses. For such a complex, heterogeneous and large genus which circumscribes diverse taxa with varying morphological characteristics, the depth of sampling is far insufficient. The intergeneric relationships between *Coelogyne* and related genera remain unclear and require further study. Some uncertainties persist and resolving the phylogenetic relationships within certain taxa remains challenging. To clarify their relationship, further investigations using additional data may depend strongly on the inclusion of *Coelogyne* in a wider geographical distribution range, particularly species occurring in the Sino-Himalaya and adjacent regions.

## Conclusion

Herein, we determined the genomic features, sequence divergences and mutation patterns in the chloroplast genomes of 24 Coelogyninae species. Comparison of genomic sequences revealed genomic variations across genera and species and provided valuable insights into the general evolutionary dynamics of Coelogyninae. Our phylogenomic analyses yielded a robust backbone phylogeny of Coelogyninae with its deep nodes well resolved. The results provide strong support for the relationships among the major groups, but also indicate non-monophyly of several genera. Although we obtained a well-supported topology consistent with earlier studies, it remains challenging to identify associated morphological characteristics in *Coelogyne* and its related genera. Thus, future studies with extensive taxon sampling and morphological evidence are needed.

## Materials and methods

### Sampling, DNA extraction and sequencing

Fresh mature leaves were plucked from the living plants cultivated at the greenhouse of South China Botanical Garden, Chinese Academy of Science (SCBG, CAS). Leaf tissues from each accession were immediately dried with silica gel for further DNA extraction. We sampled 15 species for Coelogyninae, including six *Coelogyne* species (*C. corymbosa*, *C. cristata*, *C*. *flaccida*, *C. punctulata*, *C*. *rochussenii* and *C*. *viscosa*), two *Otochilus* species (*O. fuscus* and *O. porrectus*), one *Panisea* species (*P. tricallosa*), two *Dendrochilus* species (*D*. *apoense* and *D*. *cootesii*), one *Chelonistele* species (*Ch*. *sulphurea*), representatives of monotypic *Bulleyia* (*B. yunnanensis*), Ischnogyne (*I. mandarinorum*) and *Neogyna* (*N. gardneriana*), respectively. The following cp. genome sequences were retrieved from the NCBI database: *Bletilla striata* (accession No: MT193723), *Coelogyne barbata* (accession No: NC_050858), *Pholidota chinensis* (accession No: ON880553), *P. imbricata* (accession No: ON880554), *P. protracta* (accession No: ON880560), *P. ventricosa* (accession No: ON880561), *Pleione maculata* (accession No: MW699846), *Thunia alba* (accession No: OL809658) and *Thuniopsis cleistogama* (accession No: OL809660).

Total genomic DNA was extracted using the Trelief plant genomic DNA kit, manufactured by TsingKe Biological Technology, Beijing, China. The quality of each extracted DNA samples was evaluated by 1% agarose gel electrophoresis. Libraries were constructed with insert sizes of approximately 250 to 350 bp from the same DNA sample. These qualified libraries were sequenced on an Illumina HiSeq TM2500 platform in 150 bp paired-end format performed by Novogene Bioinformatics Technology Co., Ltd., Beijing, China. For each sample, approximately 3G raw data were generated. Clean reads were obtained by removing adapter sequences, reads containing poly-N and low-quality ones.

### Plastome assembly and annotation

All raw data were first filtered for trimming of any low-quality bases or adapter sequences by FASTP [31]. Then clean reads were used to perform a de novo assembly by NOVOPlasty v.4.2.1 [32] with the *Bletilla striata* cp. genome from NCBI (MT193723) as a reference. Reference-guided connecting was subsequently conducted using Bandage v.5.6.0 [33]. The quality of the newly assembled genomes was assessed on read level basis by aligning the trimmed raw reads to the de novo assemblies using Geneious v.9.1.8 [34]. The assembled cp. genomes were annotated using the online program DOGMA [35] and GeSeq [36], and further manually adjusted and confirmed using Geneious. Additionally, for predicting transfer RNA (tRNA) genes in genomic sequences, tRNAscan-SE v.1.31 [37] was performed with the default parameters. The circular chloroplast genome maps were drawn by OGDRAW v.1.3.1 [38]. All of the 15 newly generated Coelogyninae cp. genome sequences were deposited in the NCBI GenBank database under accession numbers OR687499–OR687513.

### Repeat sequence analyses

The simple sequence repeat (SSR) loci in the 15 newly sequenced genomes, together with nine published genomes were examined via Perl script MISA v.1.01 [39]. To facilitate SSR detection, the search criteria were set as follows: 10 minimum repeat units for mononucleotide (p1) repeats, 5 for dinucleotide (p2) repeats, 4 for trinucleotide (p3), and 3 for tetranucleotide (p4), penta-, and hexa-nucleotides, respectively. The maximum distance between two adjacent SSRs in a compound SSRs (c SSRs) was less than 100 bp according to previous reports [40] and the default parameters of the MISA software. REPuter v.2.74 program [41] was utilized to analyze long repetitive sequences including forward (F), reverse (R) complement (C), and palindromic (P) repeats in the 24 Coelogyninae plastomes. The minimal repeat size was set to 30 bp long per repeat unit with Hamming distance of 3 bp.

### Plastome comparison and sequence divergence analyses

Chloroplast genome similarity was assessed using BLAST Atlas on the GView Server (https://server.gview.ca/) with 100 bp connection windows [42] with *Thuniopsis cleistogama* OL809660 as a reference. The position changes in the IR/SC junction and their adjacent genes of these cp. genomes were assessed and compared using the IRscope online program [43]. For identifying hypervariable regions, the whole-genome alignment was visualized using Shuffle-LAGAN mode [44] included in mVISTA v.2.0 [45]. Nucleotide diversity (Pi) values were calculated by DnaSP v6.12.03 software [46] with a sliding window analysis. The window length was set to 600 bp with a step size of 200 bp.

### Positive selective tests

Non-synonymous (dN) and synonymous (dS) nucleotide substitution rates, as well as their ratios (ω = dN/dS) were analyzed using CODEML program from the PAML package v.4.8a to detect sites under diversifying selection [27, 47]. The codon substitution model enables to estimate the selective pressures on protein-coding regions across divergent lineages via comparing their ratios. The ω ratio measures the mode of natural selection acting on the protein genes, with ω > 1 indicating positive (adaptive) selection, ω = 1 indicating neutral evolution, while ω < 1 signifying negative (purifying) selection. Bayes Empirical Bayes (BEB) inferences [48] were used to assess the statistical significance of potential positively selected sites. In the BEB analysis, posterior probability higher than 0.95 and 0.99 indicated sites that were under positive selection and strong positive selection, respectively (Additional file 7: Table S6).

### Phylogenetic analyses

To infer the phylogenetic relationships within Coelogyninae, we performed a series of phylogenetic analyses using both maximum likelihood (ML) and Bayesian inference (BI) methods based on two datasets (complete plastome sequences and CDS sequences). A total of 56 accessions from 42 species of 13 genera (*Bletilla*, *Bulleyia*, *Chelonistele*, *Coelogyne*, *Dendrochilus*, *Ischnogyne*, *Neogyna*, *Otochilus*, *Panisea*, *Pholidota*, *Pleione*, *Thunia* and *Thuniopsis*) representing the main lineages of Coelogyninae were included, plus one outgroup species. We were unable to obtain the material of *Aglossorrhyncha*, *Dilochia*, and *Glomera*, which were not included in the analyses. All the plastome sequences were aligned using MAFFT [49] and adjusted manually by BioEdit [50]. The ML tree was generated using FastTree 2 [51] and implemented in RAxML v.8.2.11 [52] under the generalized time-reversible GTR + G model. Nodes were evaluated by Shimodaira–Hasegawa (SH) tests [53] to detect significant topology. BI analyses of phylogeny were performed in MrBayes v.3.2.7 [54]. The Markov Chain Monte Carlo (MCMC) analyses were run for 10 million generations, employing TVM + F + R3 as the optimal model, as determined by ModelTest-NG 0.1.6 [55]. These trees were sampled every 1,000 generations, with a burn-in of 25% and the remaining trees were used to generate a majority-rule consensus tree and calculate the probability of each branch of the posterior probability (PP).

### Electronic supplementary material

Below is the link to the electronic supplementary material.

**Additional file 1:** **Figure S1.** Plastome structures of the 24 Coelogyninae species

**Additional file 2: Table S1.** Summary of major characteristics of the 24 Coelogyninae chloroplast genomes

**Additional file 3: Table S2.** Types and numbers of SSRs detected in 24 Coelogyninae species

**Additional file 4: Table S3.** Length of SSRs in the 24 Coelogyninae chloroplast genomes

**Additional file 5: Table S4.** Distribution of SSRs in the 24 Coelogyninae chloroplast genomes

**Additional file 6: Table S5.** The number of four long repeat types in the 24 Coelogyninae chloroplast genomes

**Additional file 7: Table S6.** Positive selective amino acid loci and estimation of parameters for 81 genes in subtribe Coelogyninae

**Additional file 8: Figure S2.** Bayesian (BI) phylogenetic tree of Coelogyninae using the complete chloroplast genome data. Numbers at each node are posterior probability (PP). Clades discussed in the text are labeled with clade names and indicated in colors

**Additional file 9: Figure S3.** Bayesian (BI) phylogenetic tree of Coelogyninae using protein-coding DNA sequences (CDS). Numbers at each node are posterior probability (PP). Clades discussed in the text are labeled with clade names and indicated in colors

**Additional file 10: Figure S4.** Majority-rule consensus tree of Coelogyninae derived from maximum likelihood (ML) analysis of protein-coding DNA sequences (CDS). Numbers at each node are ML bootstrap values (BS). Clades discussed in the text are labeled with clade names and indicated in colors

## Data Availability

All the newly sequenced sequences in this study have been submitted to the NCBI database (https://www.ncbi.nlm.nih.gov/genbank/) with GenBank accession numbers shown in Table S1 (OR687499-ON687513). Submitted data will remain private until related manuscript has been accepted. All data generated or analyzed are included within the article and the supplementary information files.

## Abbreviations

BI: Bayesian Inference
BLAST: Basic local alignment search tool
bp: Base pairs
CDS: Protein-coding DNA sequence
cp: Chloroplast
di-: Dinucleotides
dN: Non synonymous
DnaSP: DNA sequences polymorphism
ds: Synonymous
GC: Guanine-cytosine
IGS: Intergenic spacer
IRs: Inverted repeat
IRA: Inverted repeat A
IRB: Inverted repeat B
IRs: Inverted repeat regions
ITS: Internal transcribed spacer of ribosomal DNA
LSC: Large single copy
ML: Maximum likelihood
mono-: Mononucleotides
NCBI: National Center for Biotechnology Information
OGDRAW: OrganellarGenomeDRAW
PCG: Protein-coding gene
Pi: Nucleotide diversity
rRNAs: Ribosomal RNAs
SSC: Small single copy region
SSRs: Simple sequence repeats
tetra-: Tetranucleotides
tri-: Trinucleotides
tRNAs: Transfer RNAs

## References

[1] Freudenstein JV, Chase MW (2015). Phylogenetic relationships in Epidendroideae (Orchidaceae), one of the great flowering plant radiations: Progressive specialization and diversification. Ann Bot.

[2] Chase MW, Cameron KM, Freudenstein JV, Pridgeon AM, Salazar G, van den Berg C, Schuiteman A (2015). An updated classification of Orchidaceae. Bot J Linn Soc.

[3] Lindley J. Orchidearum sceletos. R. Taylor, London. 1826.

[4] Pridgeon AM, Cribb PJ, Chase MW, Rasmussen FN (2005). Genera Orchidacearum, volume 4. Epidendroideae (Part one).

[5] Li L, Ye DP, Niu M, Yan HF, Wen TL, Li SJ (2015). *Thuniopsis*: a new orchid genus and phylogeny of the tribe Arethuseae (Orchidaceae). PLoS ONE.

[6] Wati RK, de Graaf EF, Bogarín D, Heijungs R, van Vugt R, Smets EF, Gravendeel B (2021). Antimicrobial activity of necklace orchids is phylogenetically clustered and can be predicted with a biological response method. Front Pharmacol.

[7] Gravendeel B, Chase MW, de Vogel EF, Roos MC, Mes THM, Bachmann K (2001). Molecular phylogeny of Coelogyne (Epidendroideae; Orchidaceae) based on plastid RFLPs, *matK*, and nuclear ribosomal ITS sequences: evidence for polyphyly. Am J Bot.

[8] Chase MW, Gravendeel B, Sulistyo BP, Wati RK, Schuiteman A. Expansion of the orchid genus Coelogyne (Arethuseae; Epidendroideae) to include Bracisepalum, Bulleyia, Chelonistele, Dendrochilum, Dickasonia, Entomophobia, Geesinkorchis, Gynoglottis, Ischnogyne, Nabaluia, Neogyna, Otochilus, Panisea and Pholidota. Phytotaxa 2021;510(2): 94–134.

[9] Dong WL, Wang RN, Zhang NY, Fan WB, Fang MF, Li ZH (2018). Molecular evolution of chloroplast genomes of orchid species: insights into phylogenetic relationship and adaptive evolution. Int J Mol Sci.

[10] Guo YY, Yang JX, Bai MZ, Zhang GQ, Liu ZJ (2021). The chloroplast genome evolution of Venus slipper (*Paphiopedilum*): IR expansion, SSC contraction, and highly rearranged SSC regions. BMC Plant Biol.

[11] Han S, Wang R, Hong X, Wu C, Zhang S, Kan X (2022). Plastomes of *Bletilla* (Orchidaceae) and phylogenetic implications. Int J Mol Sci.

[12] Li L, Wu QP, Fang L, Wu KL, Li MZ, Zeng SJ (2022). Comparative chloroplast genomics and phylogenetic analysis of *Thuniopsis* and closely related genera within Coelogyninae (Orchidaceae). Front Genet.

[13] Li L, Wang WY, Zhang GQ, Wu KL, Fang L, Li MZ (2023). Comparative analyses and phylogenetic relationships of thirteen *Pholidota* species (Orchidaceae) inferred from complete chloroplast genomes. BMC Plant Biol.

[14] Qian J, Song J, Gao H, Zhu Y, Xu J, Pang X (2013). The complete chloroplast genome sequence of the medicinal plant *Salvia miltiorrhiza*. PLoS ONE.

[15] Kim KJ, Lee HL (2004). Complete chloroplast genome sequences from Korean ginseng (*Panax schinseng Nees*) and comparative analysis of sequence evolution among 17 vascular plants. DNA Res.

[16] Davis JI, Soreng RJ (2010). Migration of endpoints of two genes relative to boundaries between regions of the plastid genome in the grass family (Poaceae). Am J Bot.

[17] Huang H, Shi C, Liu Y, Mao SY, Gao LZ (2014). Thirteen *Camellia* chloroplast genome sequences determined by high-throughput sequencing: genome structure and phylogenetic relationships. BMC Evol Biol.

[18] Li DM, Li J, Wang DR, Xu YC, Zhu GF (2021). Molecular evolution of chloroplast genomes in subfamily Zingiberoideae (Zingiberaceae). BMC Plant Biol.

[19] Downie SR, Jansen RK (2015). A comparative analysis of whole plastid genomes from the Apiales: expansion and contraction of the inverted repeat, mitochondrial to plastid transfer of DNA, and identification of highly divergent noncoding regions. Syst Bot.

[20] Zhu A, Guo W, Gupta S, Fan W, Mower JP (2016). Evolutionary dynamics of the plastid inverted repeat: the effects of expansion, contraction, and loss on substitution rates. New Phytol.

[21] Ellegren H (2004). Microsatellites: simple sequences with complex evolution. Nat Rev Genet.

[22] Carmona A, Friero E, de Bustos A, Jouve N, Cuadrado A (2013). Cytogenetic diversity of SSR motifs within and between Hordeum species carrying the H genome: H. Vulgare L. and H. Bulbosum L. Theor Appl Genet.

[23] Khakhlova O, Bock R (2006). Elimination of deleterious mutations in plastid genomes by gene conversion. Plant J.

[24] Gao X, Zhang X, Meng HH, Li J, Zhang D, Liu CN (2018). Comparative chloroplast genomes of *Paris* sect. *Marmorata*: insights into repeat regions and evolutionary implications. BMC Genomics.

[25] Clegg MT, Gaut BS, Learn GH, Morton BR (1994). Rates and patterns of chloroplast DNA evolution. Proc Nati Acad Sci USA.

[26] Yang ZH, Nielsen R (2002). Codon-substitution models for detecting molecular adaptation at individual sites along specific lineages. Mol Biol Evol.

[27] Zotz G, Wilhelm K, Becker A (2011). Heteroblasty – a review. Bot Rev.

[28] Goldman DH, Freudenstein JV, Kores PJ, Molvray M, Jarrell DC, Whitten WM (2001). Phylogenetics of Arethuseae (Orchidaceae) based on plastid matK and rbcL sequences. Syst Bot.

[29] van den Berg C, Goldman DH, Freudenstein JV, Pridgeon AM, Cameron KM, Chase MW (2005). An overview of the phylogenetic relationships within Epidendroideae inferred from multiple DNA regions and recircumscription of Epidendreae and Arethuseae (Orchidaceae). Am J Bot.

[30] Huang WC, Liu ZJ, Jiang K, Luo YB, Jin XH, Zhang Z (2022). Phylogenetic analysis and character evolution of tribe Arethuseae (Orchidaceae) reveal a new genus *Mengzia*. Mol Biol Evol.

[31] Chen S, Zhou Y, Chen Y, Gu J (2018). Fastp: an ultra-fast all-in-one FASTQ preprocessor. Bioinformatics.

[32] Dierckxsens N, Mardulyn P, Smits G (2017). NOVOPlasty: de novo assembly of organelle genomes from whole genome data. Nucleic Acids Res.

[33] Wick RR, Schultz MB, Zobel J, Holt KE, Bandage (2015). Interactive visualization of *de novo* genome assemblies. Bioinformatics.

[34] Kearse M, Moir R, Wilson A, Stones-Havas S, Cheung M, Sturrock S (2012). Geneious basic: an integrated and extendable desktop software platform for the organization and analysis of sequence data. Bioinformatics.

[35] Shi LC, Chen HM, Jiang M, Wang LQ, Wu X, Huang LF, Liu C (2019). CPGAVAS2, an integrated plastome sequence annotator and analyzer. Nucleic Acids Res.

[36] Tillich M, Lehwark P, Pellizzer T, Ulbricht-Jones ES, Fischer A, Bock R, Stephan G (2017). GeSeq - versatile and accurate annotation of organelle genomes. Nucleic Acids Res.

[37] Chan PP, Lowe TM (2019). tRNAscan-SE: searching for tRNA genes in genomic sequences. Methods Mol Biol.

[38] Greiner S, Lehwark P, Bock R (2019). OrganellarGenomeDRAW (OGDRAW) version 1.3.1: expanded toolkit for the graphical visualization of organellar genomes. Nucleic Acids Res.

[39] Beier S, Thiel T, Münch T, Scholz U, Mascher M (2017). MISA-web: a web server for microsatellite prediction. Bioinformatics.

[40] Gao Z, Wu J, Liu ZA, Wang LS, Ren HX, Shu QY (2013). Rapid microsatellite development for tree peony and its implications. BMC Genomics.

[41] Kurtz S, Choudhuri JV, Ohlebusch E, Schleiermacher C, Stoye J, Giegerich R (2001). REPuter: the manifold applications of repeat analysis on a genomic scale. Nucleic Acids Res.

[42] Petkau A, Stuart-Edwards M, Stothard P, van Domselaar G (2010). Interactive microbial genome visualization with GView. Bioinformatics.

[43] Amiryousefi A, Hyvönen J, Poczai P (2018). IRscope: an online program to visualize the junction sites of chloroplast genomes. Bioinformatics.

[44] Brudno M, Do CB, Cooper GM, Kim MF, Davydov E, Green ED, Sidow A, Batzoglou S, Program NCS (2003). LAGAN and Multi-LAGAN: efficient tools for large-scale multiple alignment of genomic DNA. Genome Res.

[45] Frazer KA, Pachter L, Poliakov A, Rubin EM, Dubchak I (2004). VISTA: computational tools for comparative genomics. Nucleic Acids Res.

[46] Rozas J, Ferrer-Mata A, Sánchez-DelBarrio JC, Guirao-Rico S, Librado P, Ramos-Onsins SE (2017). DnaSP 6: DNA sequence polymorphism analysis of large data sets. Mol Biol Evol.

[47] Yang ZH (2007). PAML 4: phylogenetic analysis by maximum likelihood. Mol Biol Evol.

[48] Yang ZH, Wong WSW, Nielsen R (2005). Bayes empirical Bayes inference of amino acids sites under positive selection. Mol Biol Evol.

[49] Katoh K, Standley DM (2013). MAFFT multiple sequence alignment software version 7: improvements in performance and usability. Mol Biol Evol.

[50] Hall TA, BioEdit (1999). A user-friendly biological sequence alignment editor and analysis program for Windows 95/98/NT. Nucleic Acids Symp Ser.

[51] Price MN, Dehal PS, Arkin AP (2010). FastTree 2–approximately maximum-likelihood trees for large alignments. PLoS ONE.

[52] Stamatakis A (2014). RAxML version 8: a tool for phylogenetic analysis and post-analysis of large phylogenies. Bioinformatics.

[53] Shimodaira H, Hasegawa M (1999). Multiple comparisons of log-likelihoods with applications to phylogenetic inference. Mol Biol Evol.

[54] Ronquist F, Teslenko M, Van Der Mark P, Ayres DL, Darling A, Höhna S (2012). MrBayes 3.2: efficient bayesian phylogenetic inference and model choice across a large model space. Syst Biol.

[55] Darriba D, Posada D, Kozlov AM, Stamatakis A, Morel B, Flouri T (2019). ModelTest-NG: a New and Scalable Tool for the selection of DNA and protein evolutionary models. Mol Biol Evol.

