# Supplementary figures and images for: Comparative chloroplast genomics of 24 species shed light on the genome evolution and phylogeny of subtribe Coelogyninae (Orchidaceae)
Source: BMC Plant Biol. 2024 Jan 5;24:31. doi: 10.1186/s12870-023-04665-2 (PMC10768429; doi:10.1186/s12870-023-04665-2)

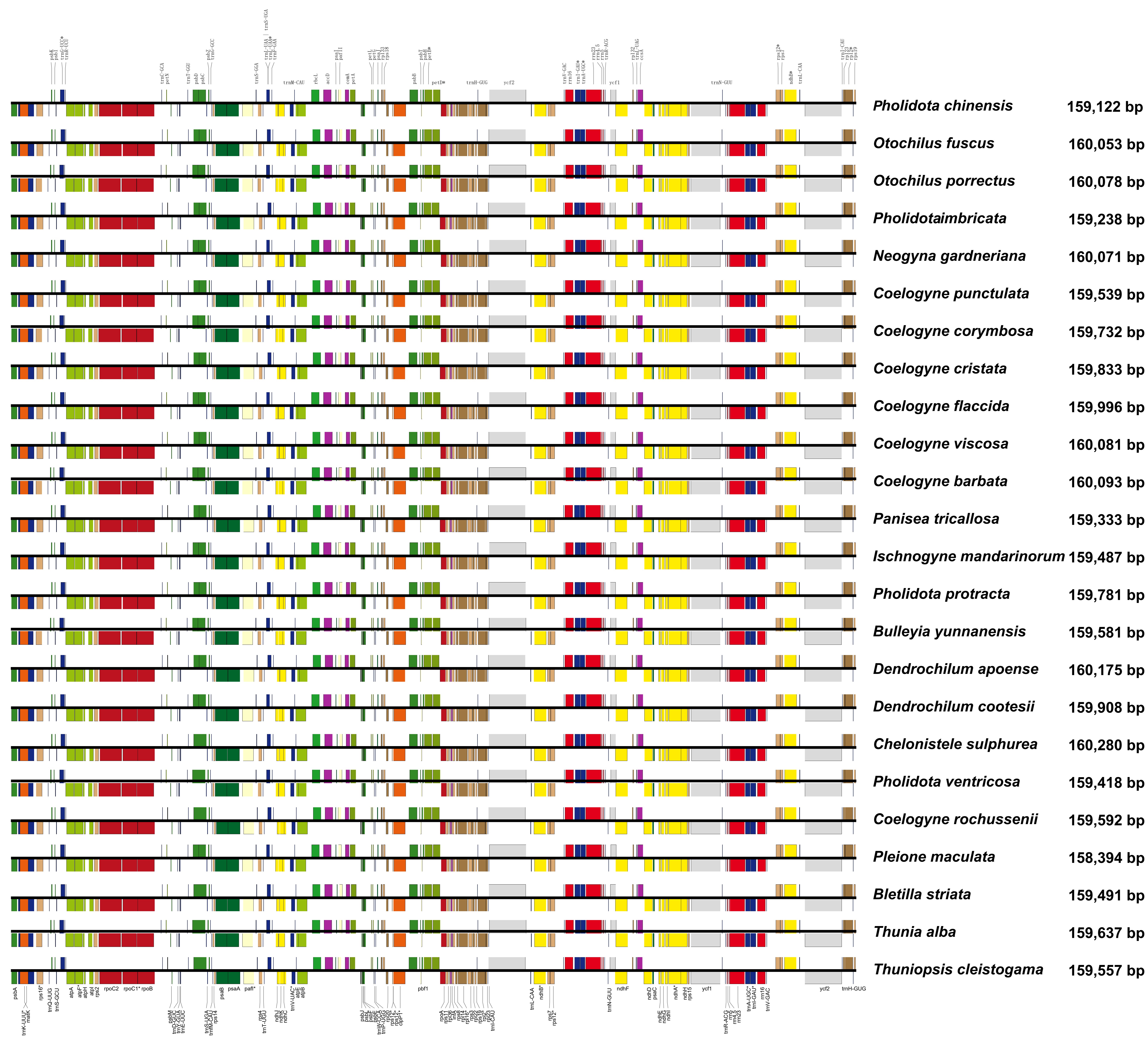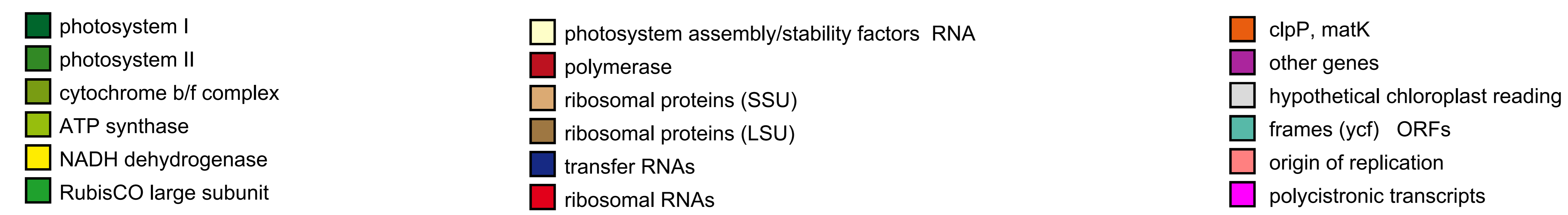

Supplement: Supplementary file 1 — Additional file 1: Figure S1. Plastome structures of the 24 Coelogyninae species [file 12870_2023_4665_MOESM1_ESM.pdf]

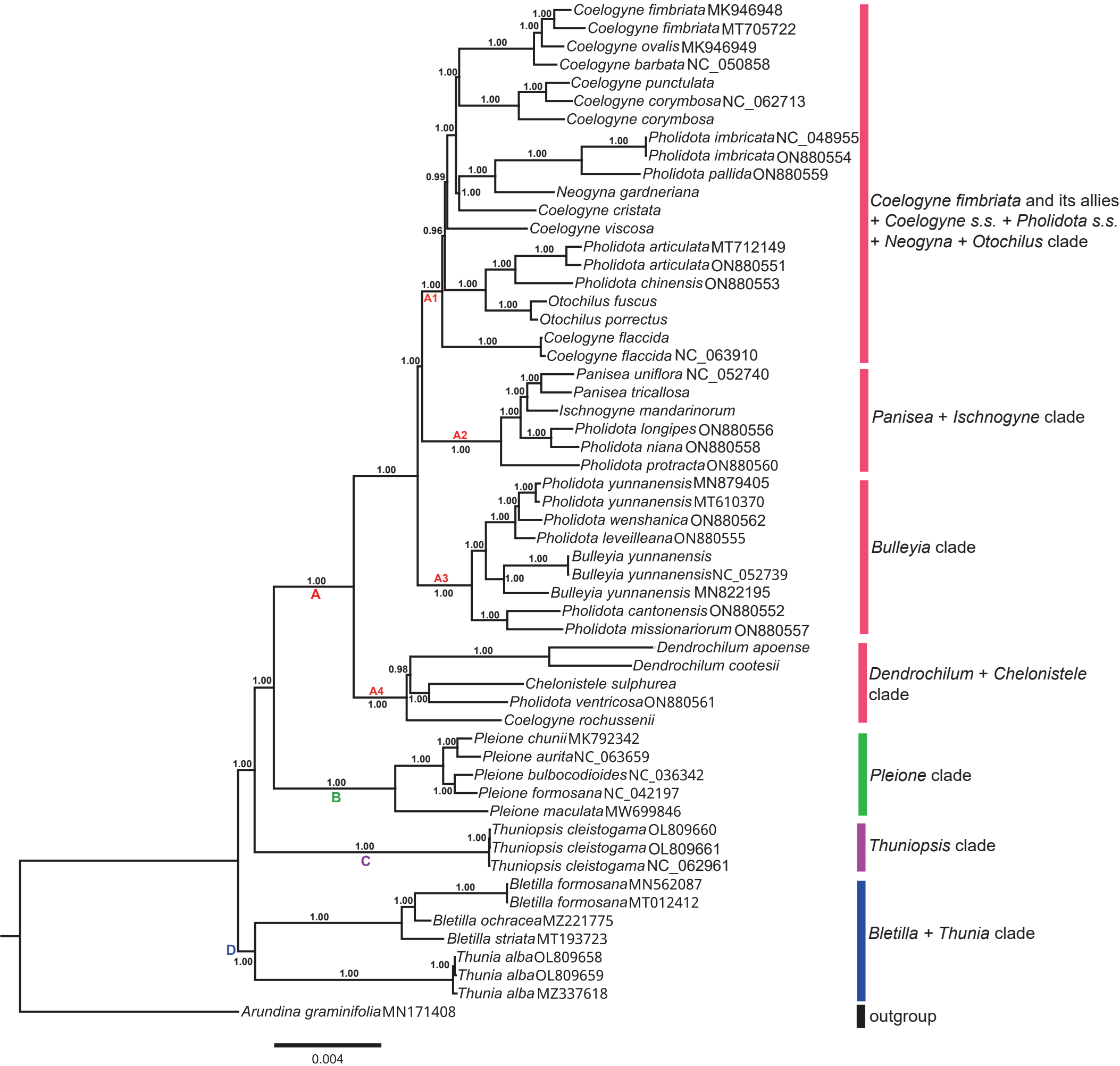

Supplement: Supplementary file 8 — Additional file 8: Figure S2. Bayesian (BI) phylogenetic tree of Coelogyninae using the complete chloroplast genome data. Numbers at each node are posterior probability (PP). Clades discussed in the text are labeled with clade names and indicated in colors [file 12870_2023_4665_MOESM8_ESM.pdf]

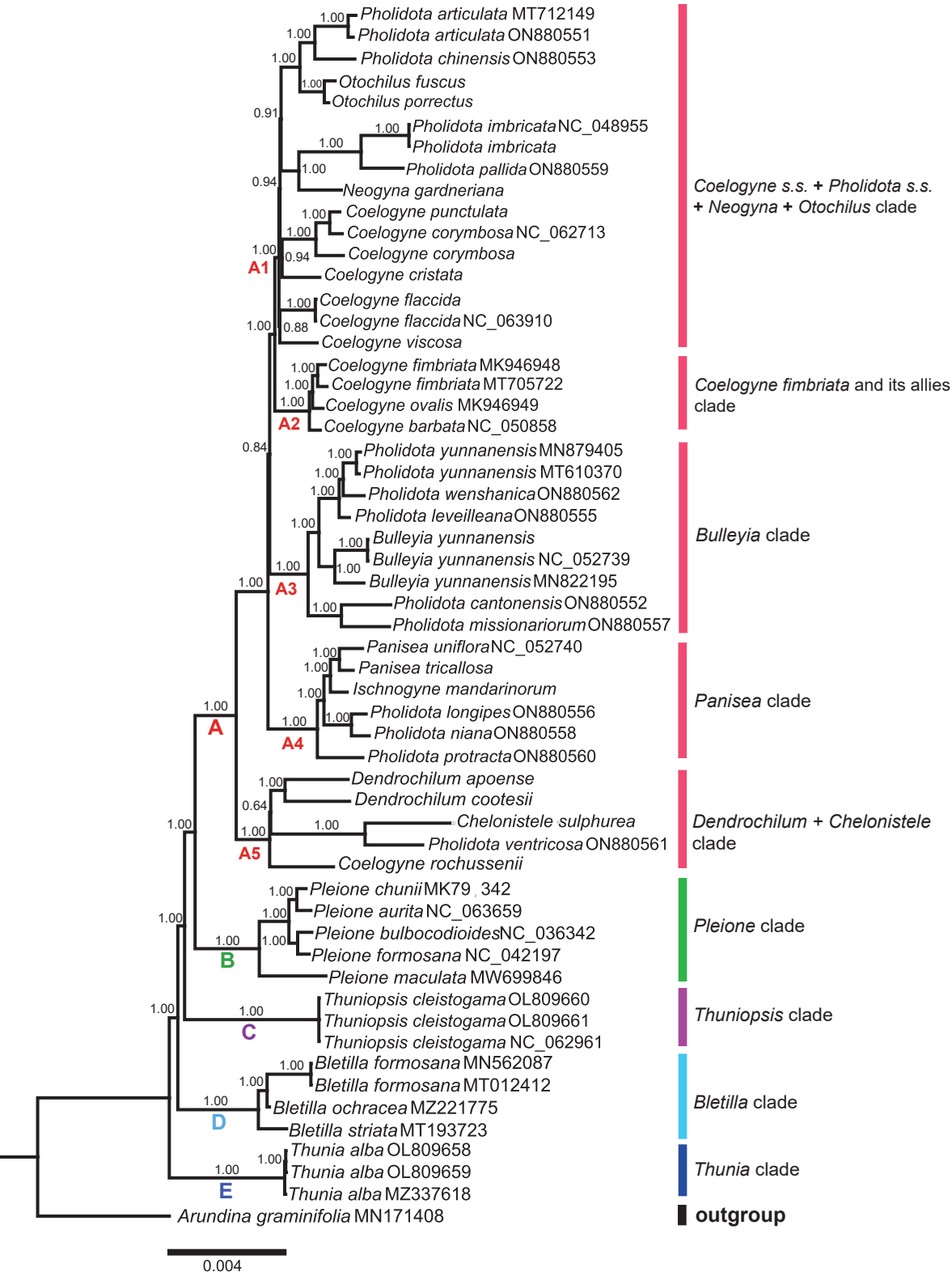

Supplement: Supplementary file 9 — Additional file 9: Figure S3. Bayesian (BI) phylogenetic tree of Coelogyninae using protein-coding DNA sequences (CDS). Numbers at each node are posterior probability (PP). Clades discussed in the text are labeled with clade names and indicated in colors [file 12870_2023_4665_MOESM9_ESM.pdf]

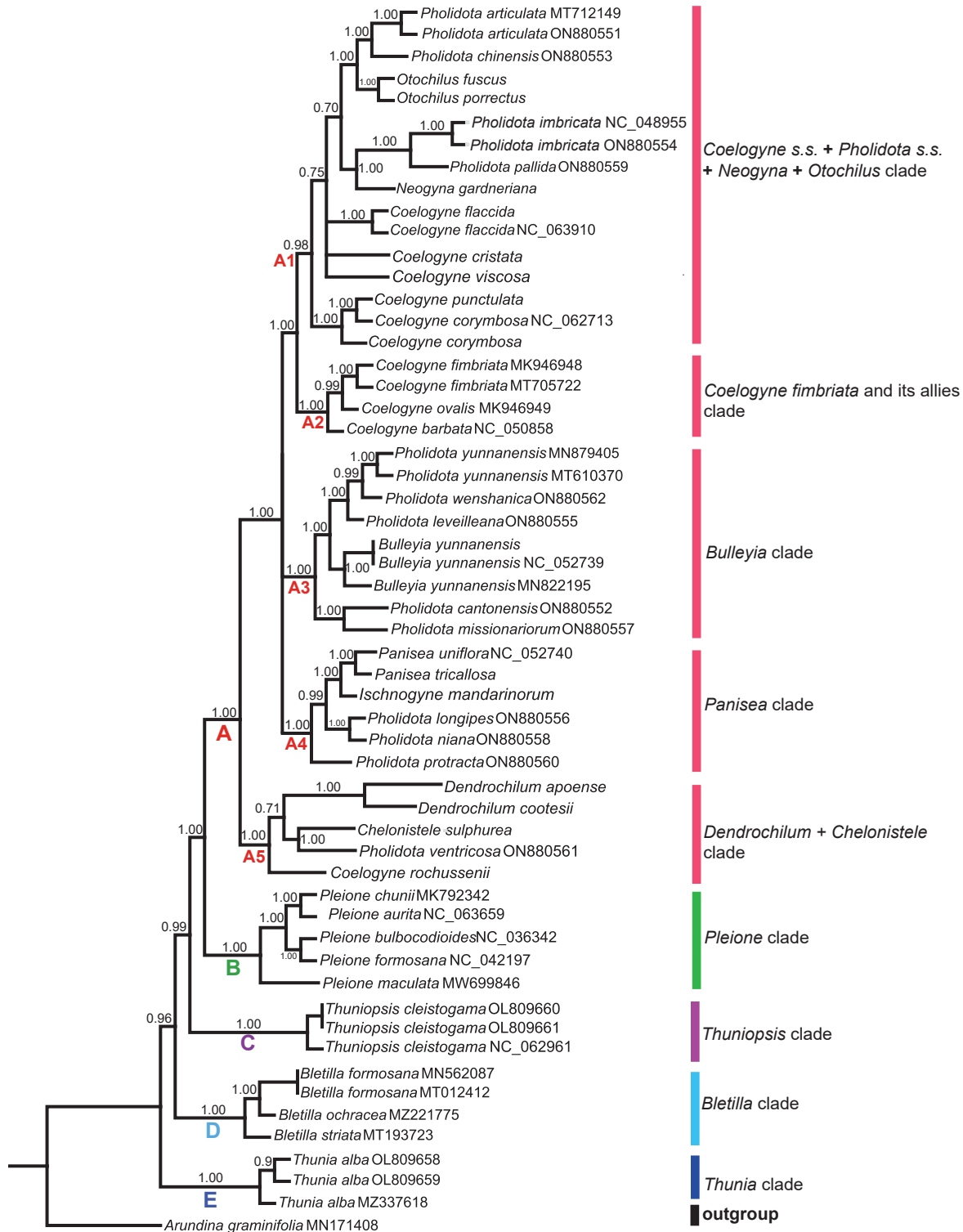

Supplement: Supplementary file 10 — Additional file 10: Figure S4. Majority-rule consensus tree of Coelogyninae derived from maximum likelihood (ML) analysis of protein-coding DNA sequences (CDS). Numbers at each node are ML bootstrap values (BS). Clades discussed in the text are labeled with clade names and indicated in colors [file 12870_2023_4665_MOESM10_ESM.pdf]
