# Supplementary material for: Comparative chloroplast genomics of 24 species shed light on the genome evolution and phylogeny of subtribe Coelogyninae (Orchidaceae)
Source: BMC Plant Biol. 2024 Jan 5;24:31. doi: 10.1186/s12870-023-04665-2 (PMC10768429; doi:10.1186/s12870-023-04665-2)
Supplement: Supplementary file 2 — Additional file 2: Table S1. Summary of major characteristics of the 24 Coelogyninae chloroplast genomes [file 12870_2023_4665_MOESM2_ESM.docx]

**Table S1 Summary of major characteristics of the 24 Coelogyninae chloroplast genomes.**

| **Species** | **Accession**  **number** | **Genome size (bp)** | **LSC** | | **SSC** | | **IR** | | **GC content**  **(%)** | **CDS genes** | **tRNA**  **genes** | **rRNA genes** |
| --- | --- | --- | --- | --- | --- | --- | --- | --- | --- | --- | --- | --- |
|  |  |  | **length (bp)** | **GC (%)** | **length (bp)** | **GC (%)** | **length (bp)** | **GC (%)** |  |  |  |  |
| *Bletilla striata* | MT193723 | 159,491 | 87,139 | 34.96 | 18,778 | 30.22 | 26,787 | 43.2 | 37.17 | 89 | 38 | 8 |
| *Bulleyia yunnanensis** | OR687500 | 159,581 | 87,563 | 35.24 | 18,720 | 30.44 | 26,649 | 43.29 | 37.37 | 89 | 38 | 8 |
| *Chelonistele* *sulphurea** | OR687501 | 160,280 | 87,856 | 35.08 | 18,832 | 30.16 | 26,796 | 43.26 | 37.24 | 89 | 38 | 8 |
| *Coelogyne barbata* | NC_050858 | 160,093 | 87,868 | 35.2 | 18,805 | 30.5 | 26,710 | 43.3 | 37.3 | 89 | 38 | 8 |
| *Coelogyne corymbosa** | OR687502 | 159,732 | 87,619 | 35.26 | 18,653 | 30.58 | 26,730 | 43.25 | 37.39 | 89 | 38 | 8 |
| *Coelogyne cristata** | OR687503 | 159,833 | 87,556 | 35.24 | 18,819 | 30.32 | 26,726 | 43.3 | 37.36 | 89 | 38 | 8 |
| *Coelogyne flaccida** | OR687504 | 159,996 | 87,647 | 35.27 | 18,885 | 30.45 | 26,732 | 43.3 | 37.39 | 89 | 38 | 8 |
| *Coelogyne punculata** | OR687505 | 159,539 | 87,598 | 35.21 | 18,703 | 30.65 | 26,619 | 43.34 | 37.39 | 89 | 38 | 8 |
| *Coelogyne rochussenii** | OR687506 | 159,592 | 87,437 | 35.1 | 18,627 | 30.31 | 26,764 | 43.25 | 37.27 | 89 | 38 | 8 |
| *Coelogyne viscosa** | OR687507 | 160,081 | 87,821 | 35.25 | 18,802 | 30.36 | 26,729 | 43.27 | 37.36 | 89 | 38 | 8 |
| *Dendrochilus apoense** | OR687508 | 160,175 | 88,206 | 34.83 | 18,615 | 29.95 | 26,677 | 43.21 | 37.05 | 89 | 38 | 8 |
| *Dendrochilus cootesii** | OR687509 | 159,908 | 87,604 | 34.91 | 18,792 | 29.9 | 26,756 | 43.24 | 37.11 | 89 | 38 | 8 |
| *Ischnogyne mandarinorum** | OR687510 | 159,487 | 87,337 | 35.12 | 18,762 | 30.15 | 26,694 | 43.28 | 37.26 | 89 | 38 | 8 |
| *Neogyna gardneriana** | OR687511 | 160,071 | 87,881 | 35.2 | 18,852 | 30.29 | 26,669 | 43.3 | 37.32 | 89 | 38 | 8 |
| *Otochilus fuscus** | OR687512 | 160,053 | 88,012 | 35.17 | 18,639 | 30.57 | 26,701 | 43.27 | 37.34 | 89 | 38 | 8 |
| *Otochilus porrectus** | OR687513 | 160,078 | 87,976 | 35.17 | 18,642 | 30.56 | 26,730 | 43.24 | 37.33 | 89 | 38 | 8 |
| *Panisea tricallosa** | OR687499 | 159,333 | 87,189 | 35.19 | 18,792 | 30.12 | 26,676 | 43.26 | 37.29 | 89 | 38 | 8 |
| *Pholidota chinensis* | ON880553 | 159,122 | 86,905 | 35.34 | 18,809 | 30.34 | 26,704 | 43.27 | 37.41 | 89 | 38 | 8 |
| *Pholidota imbricata* | ON880554 | 159,238 | 87,454 | 35.32 | 18,806 | 30.32 | 26,489 | 43.31 | 37.39 | 89 | 38 | 8 |
| *Pholidota protracta* | ON880560 | 159,781 | 87,595 | 35.22 | 18,744 | 30.28 | 26,721 | 43.29 | 37.34 | 89 | 38 | 8 |
| *Pholidota ventricosa* | ON880561 | 159,418 | 87,408 | 35.17 | 18,598 | 30.42 | 26,706 | 43.29 | 37.34 | 89 | 38 | 8 |
| *Pleione maculata* | MW699846 | 158,394 | 86,603 | 35.18 | 18,499 | 30.41 | 26,646 | 43.21 | 37.32 | 89 | 38 | 8 |
| *Thunia alba* | OL809658 | 159,637 | 87,223 | 35.09 | 18,858 | 30.28 | 26,778 | 43.24 | 37.26 | 89 | 38 | 8 |
| *Thuniopsis cleistogama* | OL809660 | 159,557 | 87,293 | 35.09 | 18,870 | 30.19 | 26,697 | 43.18 | 37.22 | 89 | 38 | 8 |

*The newly generated plastomes
